# Identification and utilization of copy number information for correcting Hi-C contact map of cancer cell lines

**DOI:** 10.1186/s12859-020-03832-8

**Published:** 2020-11-07

**Authors:** Ahmed Ibrahim Samir Khalil, Siti Rawaidah Binte Mohammad Muzaki, Anupam Chattopadhyay, Amartya Sanyal

**Affiliations:** 1 School of Computer Science and Engineering, Nanyang Technological University, 50 Nanyang Avenue, Singapore, 639798 Singapore; 2 School of Biological Sciences, Nanyang Technological University, 60 Nanyang Drive, Singapore, 637551 Singapore

**Keywords:** Chromosome conformation capture (3C), Hi-C normalization tool, Read depth, Copy number variation (CNV), Generalized linear model (GLM), Poisson regression, Cancer

## Abstract

**Background:**

Hi-C and its variant techniques have been developed to capture the spatial organization of chromatin. Normalization of Hi-C contact map is essential for accurate modeling and interpretation of high-throughput chromatin conformation capture (3C) experiments. Hi-C correction tools were originally developed to normalize systematic biases of karyotypically normal cell lines. However, a vast majority of available Hi-C datasets are derived from cancer cell lines that carry multi-level DNA copy number variations (CNVs). CNV regions display over- or under-representation of interaction frequencies compared to CN-neutral regions. Therefore, it is necessary to remove CNV-driven bias from chromatin interaction data of cancer cell lines to generate a euploid-equivalent contact map.

**Results:**

We developed the HiCNAtra framework to compute high-resolution CNV profiles from Hi-C or 3C-seq data of cancer cell lines and to correct chromatin contact maps from systematic biases including CNV-associated bias. First, we introduce a novel ‘entire-fragment’ counting method for better estimation of the read depth (RD) signal from Hi-C reads that recapitulates the whole-genome sequencing (WGS)-derived coverage signal. Second, HiCNAtra employs a multimodal-based hierarchical CNV calling approach, which outperformed OneD and HiNT tools, to accurately identify CNVs of cancer cell lines. Third, incorporating CNV information with other systematic biases, HiCNAtra simultaneously estimates the contribution of each bias and explicitly corrects the interaction matrix using Poisson regression. HiCNAtra normalization abolishes CNV-induced artifacts from the contact map generating a heatmap with homogeneous signal. When benchmarked against OneD, CAIC, and ICE methods using MCF7 cancer cell line, HiCNAtra-corrected heatmap achieves the least 1D signal variation without deforming the inherent chromatin interaction signal. Additionally, HiCNAtra-corrected contact frequencies have minimum correlations with each of the systematic bias sources compared to OneD’s explicit method. Visual inspection of CNV profiles and contact maps of cancer cell lines reveals that HiCNAtra is the most robust Hi-C correction tool for ameliorating CNV-induced bias.

**Conclusions:**

HiCNAtra is a Hi-C-based computational tool that provides an analytical and visualization framework for DNA copy number profiling and chromatin contact map correction of karyotypically abnormal cell lines. HiCNAtra is an open-source software implemented in MATLAB and is available at https://github.com/AISKhalil/HiCNAtra.

## Background

3C-related methods are a collection of molecular techniques to analyze three-dimensional (3D) chromatin interactions inside the cell nucleus [1]. Since its inception, the 3C technique [2] has been modified and combined with next-generation sequencing (NGS) to interrogate chromatin interactions of genomic loci at different length scales [1, 3, 4]. The genome-wide adaptation of the 3C technique, Hi-C, confirmed the chromosome territory hypothesis and revealed the hierarchical organization of our genome into A/B compartments [3] and topologically-associating domains (TADs) [5, 6]. Subsequently, the 3C-sequencing (3C-seq) technique was introduced as a simplified Hi-C method by combining conventional 3C library preparation protocol and NGS [4, 7, 8].

There is an abundance of Hi-C datasets generated using cancer cell lines. Cancer genomes generally exhibit abnormal karyotypes and carry pervasive genomic alterations, including large-scale and focal CNVs [9–13], with pathological consequences [14]. It has been reported that CNV results in the rewiring of chromatin connectivity leading to alterations in the long-range control of gene expression [15]. CNVs can directly modulate gene-regulatory mechanisms by altering the copy number (CN) of regulatory elements or indirectly by modifying the higher-order chromatin structure [16]. For example, prostate cancer cell lines have been shown to harbor smaller-sized TADs compared to the normal prostate epithelial cells, with new TAD boundaries coinciding with the CNV regions [17]. Similarly, multiple myeloma (MM) cells, with whole/partial chromosomal gains or losses, exhibit an increment of 25% in the number of TADs and a significant decrease in their average size compared to normal B cells [18]. Additionally, CN-amplified regions in MM cells manifest in higher interaction frequencies than CN-neutral (normal) regions. This is expected as regions with CN gains or losses will have relatively greater or lower chances of being captured during the Hi-C pull-down step, respectively, which in turn will affect interaction profiles of CNV regions [15, 18, 19]. Taken together, these observations provide evidence that chromatin interaction signal is adversely impacted by CNVs in cancer cell lines with abnormal karyotypes. Therefore, the apparent CNV effect on contact frequency must be masked/corrected to obtain a euploid-equivalent contact map for correct interpretation of genome-wide chromatin interaction data.

Nevertheless, for correcting the raw contact map from CNV effects, it is a prerequisite to collect information about genome-wide DNA copy number profile. Hi-C sequencing reads can be successfully utilized to estimate the RD signal and discovery of CNVs without the additional cost of performing WGS. HiCnv is the first tool to identify CNVs from Hi-C reads [20]. However, it can only identify large CNVs (> 1 Mb size). Recently, HiNT [21] has been developed for detecting CNVs of smaller sizes from Hi-C data. HiNT utilizes a generalized additive model (GAM) based on unimodal Poisson distribution, introduced earlier in BIC-seq2 [22], to normalize the read counts. This leaves plenty of room for improvement for identifying CNVs from Hi-C/3C-seq reads by using a multimodal RD signal distribution which is particularly suited for cancer cell lines [23].

Hi-C contact maps are affected by different kinds of biases [24–26]. Several Hi-C normalization procedures have been developed over the years to correct the effects of these biases, which can be broadly divided into implicit- and explicit-based methods. Implicit methods correct the Hi-C contact map as a matrix-balancing problem assuming that genomic regions binned at equal size would yield similar coverage [27–31]. On the other hand, explicit methods, that require a priori knowledge of the biases, normalize the contact matrix by modeling the relationship between the contact frequencies and the known sample-independent systematic biases introduced primarily by GC-content, effective (restriction) fragment length, and mappability biases [24, 32].

ICE (Iterative Correction and Eigenvector decomposition) method [27] has been commonly used to implicitly correct the contact maps of most Hi-C datasets without the requirement of a priori knowledge of the bias sources. Though the ICE method may indirectly account for the effects of CNV-driven bias on the interaction matrix, recent evidence demonstrated that ICE method is not optimum to correct Hi-C data of cancer cells that are plagued with widespread CNVs [26, 30]. These studies showed that ICE normalization results in overcompensation of original CNV bias leading to distortion of the interaction frequency signal. A crude attempt to normalize CNV bias was first presented by caICB, a chromosome-adjusted iterative correction method [33]. The caICB applies a uniform copy number for individual chromosomes ignoring the copious presence of segmental aneuploidy and focal CNVs. Two methods have been recently introduced to address the CNV bias effects on Hi-C contact maps. First, the explicit-based approach of OneD [26] normalizes the contact map sequentially for the sample-independent systematic biases (GC-content, effective fragment length, and mappability) followed by dividing the contact frequencies between two genomic loci by the product of their copy numbers, estimated by a Hidden Markov Model (HMM). On the other hand, the implicit-based approach of CAIC (CNV-Adjusted Iterative Correction) [30] first performs a local iterative correction. Then, it normalizes the CNV-bias by modeling the raw interaction count between two CNV loci as the product of their copy numbers, which are estimated using a pruned dynamic programming algorithm [34]. However, our analysis showed that the performance of OneD and CAIC tools is not optimum for the removal of CNV-induced artifacts from the Hi-C contact maps of cancer cell lines.

Here, we developed HiCNAtra (**Hi**-**C** **N**ormalization **A**pproach **t**hrough **r**ead depth **a**nalysis) framework, which can (1) estimate DNA CN profile and detect CNVs from Hi-C/3C-seq data and, (2) apply this CN information for normalization of chromatin contact map. HiCNAtra utilizes all types of raw Hi-C reads using a novel ‘entire-fragment’ counting approach for computing RD signal, while genomic reads are exclusively used in the case of 3C-seq data. RD signal computed by HiCNAtra from Hi-C/3C-seq data successfully recapitulates the WGS-derived RD signal of the corresponding cell line. Our RD-computing approach can extract the RD signal at high resolution that allows precise detection of both large-scale and focal CN alterations using the multimodal distribution-based hierarchical CNV calling approach of CNAtra [23]. Benchmarking of HiCNAtra’s CNV caller module against OneD and HiNT showed that HiCNAtra is the only tool to accurately estimate the CN profiles of karyotypically abnormal cell lines. For contact map normalization, HiCNAtra utilizes a generalized linear model (GLM) to correct the interaction matrix from the *sample-dependent* CNV bias along with *sample-independent* systematic biases (GC-content, effective fragment length, and mappability). We evaluated the contact-map correction performance of HiCNAtra against ICE, OneD, and CAIC methods using Hi-C data of MCF7 breast cancer cell line. Comparative results showed that the HiCNAtra-corrected contact map achieved the least 1D signal variation with no signal inversion effects, unlike implicit-based approaches. Besides, HiCNAtra normalization successfully removed the CNV-induced artifacts from the contact map which results in a heatmap with homogeneous signal. Further, head-to-head comparison of explicit-based Hi-C correction tools, HiCNAtra and OneD, using Hi-C/3C-seq datasets of five cancer cell lines established the superiority of HiCNAtra in ameliorating the effects of CNV-induced bias and other systematic biases on chromatin contact maps.

## Results

### Overview of HiCNAtra framework

**Fig. 1:**
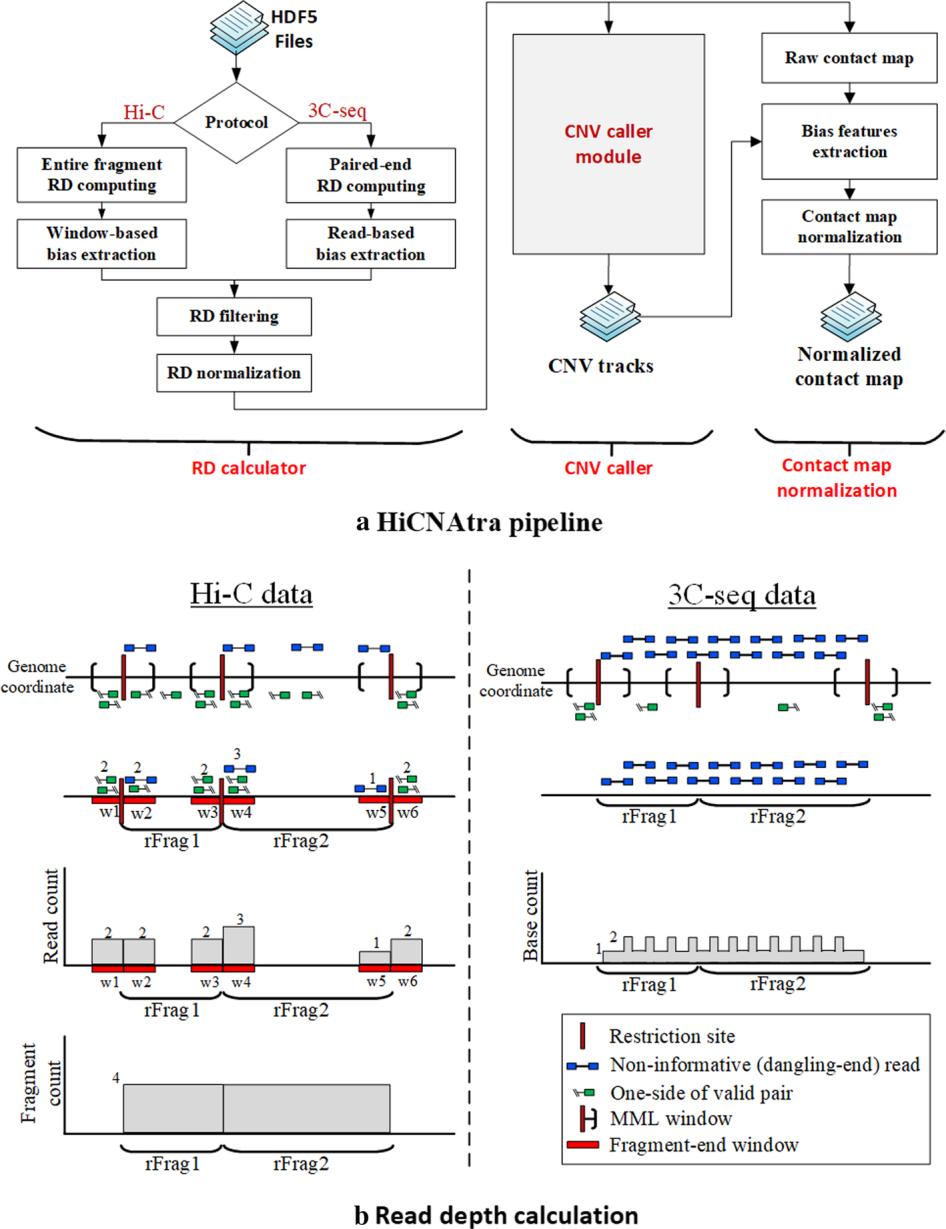
HiCNAtra pipeline and RD signal computation. **a** Block diagram of the HiCNAtra software showing different computational modules. **b** Schematic illustration of the RD signal computation methods using Hi-C (left) and 3C-seq (right) reads. Left: For Hi-C data, informative and non-informative (only dangling-end reads are shown) reads (top panel) mapped within the restriction fragment-end windows (second panel) are used for RD calculation. Then, the number of reads is counted for each window (third panel). Base counts are then estimated for each restriction fragment as the summation of the counts of its two fragment-end windows (bottom panel). Right: For 3C-seq data (top panel), genomic reads (middle panel) are exclusively used to compute the RD signal in an unbiased manner similar to WGS paired-end reads (bottom panel)

HiCNAtra pipeline comprises of three modules: RD calculator, CNV caller, and contact map normalization (Fig. 1a). The *RD calculator* module accomplishes the computation of the RD signal from NGS reads of Hi-C or 3C-seq datasets (default bin size = 5 kb). In the case of Hi-C data, both ‘informative’ as well as ‘non-informative’ reads, are utilized to compute the RD signal employing a novel entire-(restriction) fragment counting approach (Fig. 1b, left). The informative reads constitute valid pairs that represent interactions between genomic loci, whereas non-informative reads include all other types of reads (dangling-end, extra dangling-end, self-circle, and single-sided reads).

In this approach, we count the reads for each restriction fragment based on the assumption that each continuous DNA-sequence read represents a particular restriction fragment and contributes to the abundance of that fragment. So, for a particular restriction fragment, the fragment count is calculated as the sum of the number of reads located within a distance of maximum molecule length (MML) from the restriction site (see “Methods” section). In the case of 3C-seq datasets, genomic reads are distributed uniformly along the genome and represent the majority of reads (Fig. 1b, right). Therefore, the RD signal is exclusively computed from the paired-end genomic reads in an unbiased manner similar to RD analysis of paired-end WGS reads (see “Methods” section).

The *CNV caller* module identifies CNVs in cancer cell lines using the RD signal computed by the RD calculator module. It incorporates the multimodal RD signal modeling approach of CNAtra [23] to hierarchically identify large-scale and focal CN-altered segments. These CN events are integrated to generate the CNV track that is used as an explicit bias source for correcting chromatin interaction matrix.

Finally, the *contact map normalization* module utilizes a Poisson-based GLM to simultaneously normalize the contact matrix for GC-content, effective fragment length and mappability biases, as well as biases introduced by CN gains/losses. This simultaneous approach allows HiCNAtra to estimate the accurate contribution of each bias source on the contact frequencies and correct them. HiCNAtra also performs pre-processing of Hi-C/3C-seq data and generates the pre- and post-normalization chromatin contact maps for visual inspection.

### HiCNAtra computes high-resolution RD signal by utilizing all types of Hi-C reads

Data coverage (number of reads) is an important determinant for detecting CNVs, especially the focal alterations. Although valid pairs are the main target of Hi-C/3C-seq analysis for capturing the chromatin interactions, other non-informative reads can be additionally utilized for estimation of the RD signal at a higher resolution. Based on our analyses of six Hi-C datasets that we used in this study, valid pairs represent 39–64% of the total mapped reads that include single-sided and double-sided mapped reads (Additional file 2: Table S1). Other reads, which represent a significant proportion (36–61%), are usually filtered out during Hi-C downstream analysis. However, these reads are still ‘*informative*’ and can potentially contribute to the estimation of the genome-wide RD signal. Therefore, we computed the RD signal from all Hi-C reads (both informative and non-informative reads) or utilizing valid pairs only. We found that the addition of non-informative reads increases the resolution of the RD signal by 22–44% for different Hi-C datasets (Additional file 2: Table S1). In order to substantiate the validity of incorporating non-informative reads for estimating the RD signal, we compared “read counts per restriction fragment” from all Hi-C reads versus valid pairs only using the same number of randomly-selected reads (Additional file 3: Table S2). We found that read counts from all Hi-C reads and valid pairs are highly correlated (Spearman correlation 80–91%) (Additional file 3: Table S2). These findings confirm that the inclusion of non-informative Hi-C reads productively adds toward the computation of a high-resolution RD signal without introducing any undesired bias in the read counts.

On the other hand, the 3C-seq technique results in a majority of genomic reads among the double-sided mapped reads (percentage of genomic reads ranges from 66% in K562 to 77% in H69AR data). Therefore, similar to WGS datasets, RD signals can be conveniently computed from these genomic reads in an unbiased manner. Each mapped paired-end genomic read is used to calculate the fragment length (side1-start to side2-end), which helps to compute the RD signal at high coverage (14.26 × for K562 with 117 million paired-end genomic reads and 12.37 × for H69AR with 120 million paired-end genomic reads). Altogether, HiCNAtra maximally utilizes the Hi-C/3C-seq reads for computing RD signal in contrast to other Hi-C tools (HiCnv, HiNT, OneD, and CAIC) that use valid pairs only [20, 21, 26, 30].

### Entire-fragment counting approach successfully extracts copy number-associated features of RD signal

Accurate estimation of the RD signal from Hi-C reads is the primary key for the identification of CNV regions. Generally, most Hi-C analysis tools [20, 21, 24–32, 35] assign the Hi-C reads to the midpoint of the corresponding restriction fragment (*midpoint* approach) or map reads to their exact-cut coordinates (*exact-cut* approach). These approaches are suitable for interaction frequency calculation since Hi-C matrices are usually generated using a larger bin size (usually one-order of magnitude larger) compared to the average length of the restriction fragments. However, smaller bins are necessary for a better identification of change points at high-resolution and detection of CNVs of different length scales. The microscopically detectable large-scale copy number variations (LCVs) and sub-microscopic focal alterations (FAs) coexist in cancer genomes [36–38]. While LCVs are Mb-scale chromosomal abnormalities, such as segmental aneuploidy [38], FAs are CNVs of size ranging from kb to a few Mb, containing a single or few genes related to cancer or drug resistance [12, 37]. Therefore, we formulated a novel *entire-(restriction) fragment* counting method where we assign the count of mapped Hi-C reads of a particular restriction fragment to each base (nucleotide) of that fragment for RD signal calculation (Fig. 1b).

**Fig. 2:**
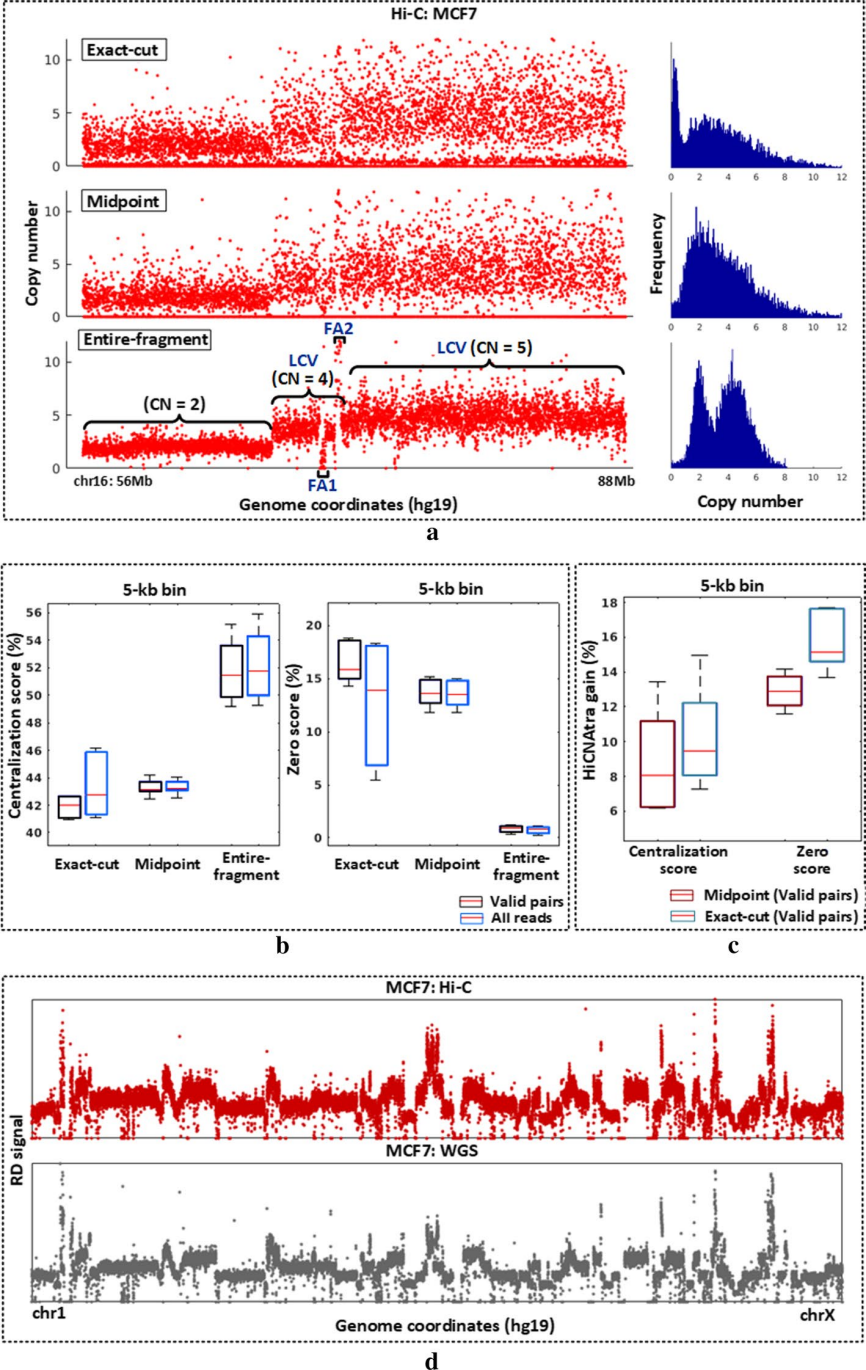
RD signal computed from entire-fragment counting approach recapitulates WGS-derived coverage signal. **a** The coverage plot (left) at 5-kb bin and the RD frequency distribution (right) of MCF7 chr16:56-88 Mb region computed from Hi-C data using exact-cut (top), midpoint (middle), and HiCNAtra’s entire-fragment (bottom) counting approaches. Each red dot represents the copy number per bin. Focal alterations (e.g. FA1 and FA2) and LCVs are shown. **b** Box plot of centralization scores (5-kb bin) and zero scores (5-kb bin) computed from the Hi-C-derived RD signal of 6 cell lines (MCF7, LNCaP, PC3, GM12878, IMR90, and PrEC) using exact-cut, midpoint, and entire-fragment counting approaches at 5-kb and 100-kb bin sizes. **c** HiCNAtra gain (in percentage) of centralization and zero scores by utilizing the entire fragment counting approach, compared to exact-cut and midpoint approaches. **d** The genome-wide coverage plot (chr 1–chr X) of MCF7 breast cancer cell line is computed from Hi-C (top) and WGS (bottom) data. Each dot represents the RD signal per bin computed from Hi-C data (red) or WGS data (grey). WGS data is obtained from the ‘input’ (control) data of MCF7 ChIP-seq experiment

For illustrating the advantage of HiCNAtra’s entire-fragment method over the exact-cut and midpoint approaches, we first visually compared their RD signals using MCF7 Hi-C data generated using the HindIII enzyme (Fig. 2a). We calculated the RD signal at 5-kb bin using Hi-C reads, which is comparable to the experimental resolution (average 4096 bases for 6-bp cutter). Interestingly, we found that the entire-fragment counting method can credibly capture LCVs in MCF7 cells as evidenced by two main peaks in the RD signal distribution (Fig. 2a right, bottom panel). Besides, FAs are visually conspicuous using our entire-fragment counting method viz*.* FA1 and FA2 in the RD signal plot (Fig. 2a left, bottom panel). In comparison, RD signals generated using the other two approaches show high signal variability and make the distribution more skewed toward bins with low reads (Fig. 2a, top and middle panels). Overdispersion can be observed in the genome-wide RD signal in both karyotypically normal lymphoblastoid cells (GM12878) and LNCaP prostate cancer cells with an abnormal karyotype (Additional file 1: Fig. S1). As a visual illustration, the RD signal of chromosome (chr) 3 (which is devoid of LCVs in both GM12878 and LNCaP), computed by the entire-fragment method, showed minimal variation level compared to other approaches as indicated by the height of the red arrow in the Additional file 1: Fig. S1.

More importantly, we examined the impact of different RD-computing approaches on the CNV calling by HiCNAtra. For that, we applied HiCNAtra’s CNV caller module on the RD signals of MCF7 and LNCaP cancer cell lines computed by the three approaches (Additional file 1: Figs. S2 and S3). Overall results showed that midpoint and exact-cut approaches failed to capture RD signal amplitude change in many cases. Considering the CNVs identified by CNAtra [23] (ref) using WGS datasets of MCF7 and LNCaP as ground truth, HiCNAtra’s CNV caller outputs using midpoint and exact-cut approaches resulted in several false negatives. For example, in the MCF7 chr 1 (80–100 Mb) region, HiCNAtra’s CNV caller output using the entire-fragment counting method revealed high concordance with the CNAtra’s WGS-derived CNV output of the same cell line. On the other hand, the midpoint and exact-cut approaches could not detect any CNV (Additional file 1: Fig. S2). It is important to mention that the CNVs derived from WGS versus Hi-C data may not be exactly superimposable because of clonal/strain variations of the cell line as well as differences in the depth of coverage of the datasets. Similarly, in the case of LNCaP chr 10 (85–120 Mb), the midpoint and exact-cut approaches detected few CNVs correctly, however, they missed/incorrectly called other CNVs (Additional file 1: Fig. S3). This discrepancy in CNV detection between exact-cut versus other counting approaches can be attributed to the visual observation that these CNVs are not conspicuous in the RD signal computed by the midpoint and exact-cut approaches.

As a quantitative comparison, we computed the centralization and zero scores for all the six Hi-C datasets (GM12878, IMR90, PrEC, MCF7, LNCaP, and PC3). The centralization score (CS) is computed as the percentage of bins that are near their integer CN states (CN = 1, 2, 3, and so on):

$$CS = \frac{{\mathop \sum \nolimits_{i} W_{i} { },{ }\left| {S_{i} - CN_{i} } \right| \le 0.25}}{{\mathop \sum \nolimits_{i} W_{i} { }}}$$

where *i* is the bin number, $CN_{i}$ is the copy number of the bin, $S_{i}$ is the CN state of the bin, and $W_{i}$ is the bin size. CS can be used as a measure for the allocation of genomic loci at or near integer CN states. Next, we defined the zero score (ZS) as the percentage of bins with CN = 0. ZS can be used as a measure of the sparseness of the RD signal. Therefore, in a nutshell, high CS implies that the RD signal is distributed near distinct CN states which aids in the identification of CNVs, while low ZS is essential to avoid false detection of deleted regions, especially in large restriction fragments. We compared the entire-fragment counting method against midpoint and exact-cut approaches using only valid read pairs as well as all Hi-C reads. In both settings, we found that the entire-fragment counting approach achieved the maximum CS and minimum ZS in all Hi-C datasets (Fig. 2b, Additional file 4: Table S3). Additionally, we found that incorporating other reads with valid pairs increases the CS and decreases the ZS with different degrees (Fig. 2b, Additional file 3: Table S3). Notably, at 5-kb binning, the exact-cut approach reaped the maximum benefit of integrating other types of Hi-C reads (mean ∆CS = 1.42% and ∆ZS = 3.6468%), compared to entire-fragment (mean ∆CS = 0.365% and ∆ZS = 0.076%) and midpoint (mean ∆CS = 0.0276% and ∆ZS = 0.129%) approaches (Fig. 2b, Additional file 3: Table S3). This suggests that other read types complement the valid pair reads as they are partially mapped to different genomic loci. The HiCNAtra gain (in percentage) of CS and ZS is computed as the absolute difference in score between the entire fragment counting approach (using all Hi-C reads, default settings) and other approaches (using valid pairs, default settings) (Fig. 2c, Additional file 5: Table S4). HiCNAtra gain of CS ranges from 6.1 to 13.4% with respect to the midpoint and 7.28–14.9% compared to the exact-cut approach. Similarly, ZS gain ranges from 11.6 to 14.2% compared to the midpoint approach, and 13.65–17.7% over the exact-cut approach. This quantitative comparison further strengthens the utility of the entire-fragment counting method and conforms to the conclusions derived from visual inspection of coverage plots and CNV profiles derived from these three approaches (Fig. 2a, Additional file 1: Figs. S1–S3).

Finally, we compared the RD signal derived from Hi-C reads with the WGS-derived coverage signal of the same cell line. We found that RD signal computed from Hi-C reads using the entire-fragment method has the highest correlation with the WGS-derived RD signal (Spearman's ρ of 0.714 for MCF7 and 0.55 for LNCaP cells) (Additional file 6: Table S5). In comparison, exact-cut and midpoint approaches showed the Spearman's ρ of 0.32 and 0.31 for MCF7, and 0.26 and 0.24 for LNCaP cells, respectively (Additional file 6: Table S5). Visual inspection of genome-wide RD signal computed by these three approaches (entire-fragment, exact-cut, and midpoint) versus the WGS-derived RD signal (Additional file 1: Fig. S4) further validates the supremacy of the entire-fragment method to correctly extract RD signal from Hi-C reads. As expected, we observed that the RD signal extracted from K562 3C-seq data correlated well with the WGS-derived coverage signal (Spearman’s ρ = 0.573) (Additional file 6: Table S5). These data indicate that the RD signal can be reproducibly extracted from Hi-C/3C-seq datasets (visually illustrated in Fig. 2d and Additional file 1: Fig. S5). Taking all results together, our analysis showed that HiCNAtra is the best estimator of the RD signal from Hi-C/3C-seq reads.

### HiCNAtra outperforms OneD and HiNT tools for identifying CNVs from Hi-C/3C-seq data of cancer cell lines

All computation tools that identify CNVs from Hi-C data, such as HiCnv, HiNT, and OneD, adapted their CNV calling algorithm from different WGS-derived CNV detection tools. HiCnv [20], the first tool developed for detecting CNV from Hi-C data, computes the RD signal (30-kb bin) from the interaction matrices. It employs an HMM-based segmentation and utilizes the global average of RD signal as CN reference to identify CNVs. However, HiCnv was tuned to identify only large-scale CNVs with size > 1 Mb. Recently, HiNT [21] has been developed for detecting CNVs of smaller sizes from Hi-C data. Similar to HiCnv, HiNT computes the RD signal as the row summation of the Hi-C interaction matrices in cooler format [35]. HiNT adapted the pipeline of BIC-seq2 [22] for identifying CNVs from the Hi-C-derived RD signal. BIC-seq2, a WGS-based CNV detection tool, inferred the CN of a genomic region as the ratio between the observed mapped reads and the expected mapped reads, estimated using Poisson-based GAM, in that region. On the other hand, OneD’s CNV detection module utilizes an HMM of eight states to estimate the copy number of genomic segments [26]. Therefore, all CNVs with CN ≥ 8 are assigned the same state which may impact the CNV-driven bias correction of regions with high copy number gain.

HiCNAtra’s CNV caller module adapted the CNAtra approach [23] to identify CNVs. CNAtra introduced a novel hierarchical CNV detection method based on multimodal distribution to identify both LCVs and FAs in cancer cell lines regardless of the complexity of structural variations. We found that RD frequency distributions of cancer cell lines (MCF7, LNCaP, PC3, H69AR, and K562) derived from Hi-C/3C-seq reads exhibited multimodal distribution (Additional file 1: Fig. S6a), whereas normal cell lines (GM12878, IMR90, and PrEC) showed unimodal RD frequency distribution of (Additional file 1: Fig. S6b). Additionally, Hi-C/3C-seq experiments are performed on cancer cell lines which generally do not have normal counterparts to serve as the reference controls. Hence, we adapted the CNAtra approach which uses a single sequencing sample as input to identify CNVs in cell lines with abnormal karyotypes. This approach is also applicable to karyotypically normal cell lines exhibiting unimodal RD signal distributions since unimodal is a special form of multimodal distribution.

After successful extraction of RD signals from Hi-C/3C-seq reads that are consistent with the WGS-derived RD signal, we analyzed the CNV profiles of six Hi-C and two 3C-seq datasets using the CNV caller module of HiCNAtra (Additional file 7: Table S6). Results show that all cancer cell lines (MCF7, LNCaP, PC3, K562, and H69AR) are enriched for LCVs (45–131 regions) with a median width of 5.2–38.9 Mb, whereas normal cell lines (GM12878, IMR90 and PrEC) are almost free of LCVs (1–3 regions) (Additional file 1: Fig. S7a). On the other hand, FAs are pervasive in both cancer (172–259 regions) and normal cell lines (21–102 regions) with median width of 155–235 kb (Additional file 1: Fig. S7b).

**Fig. 3:**
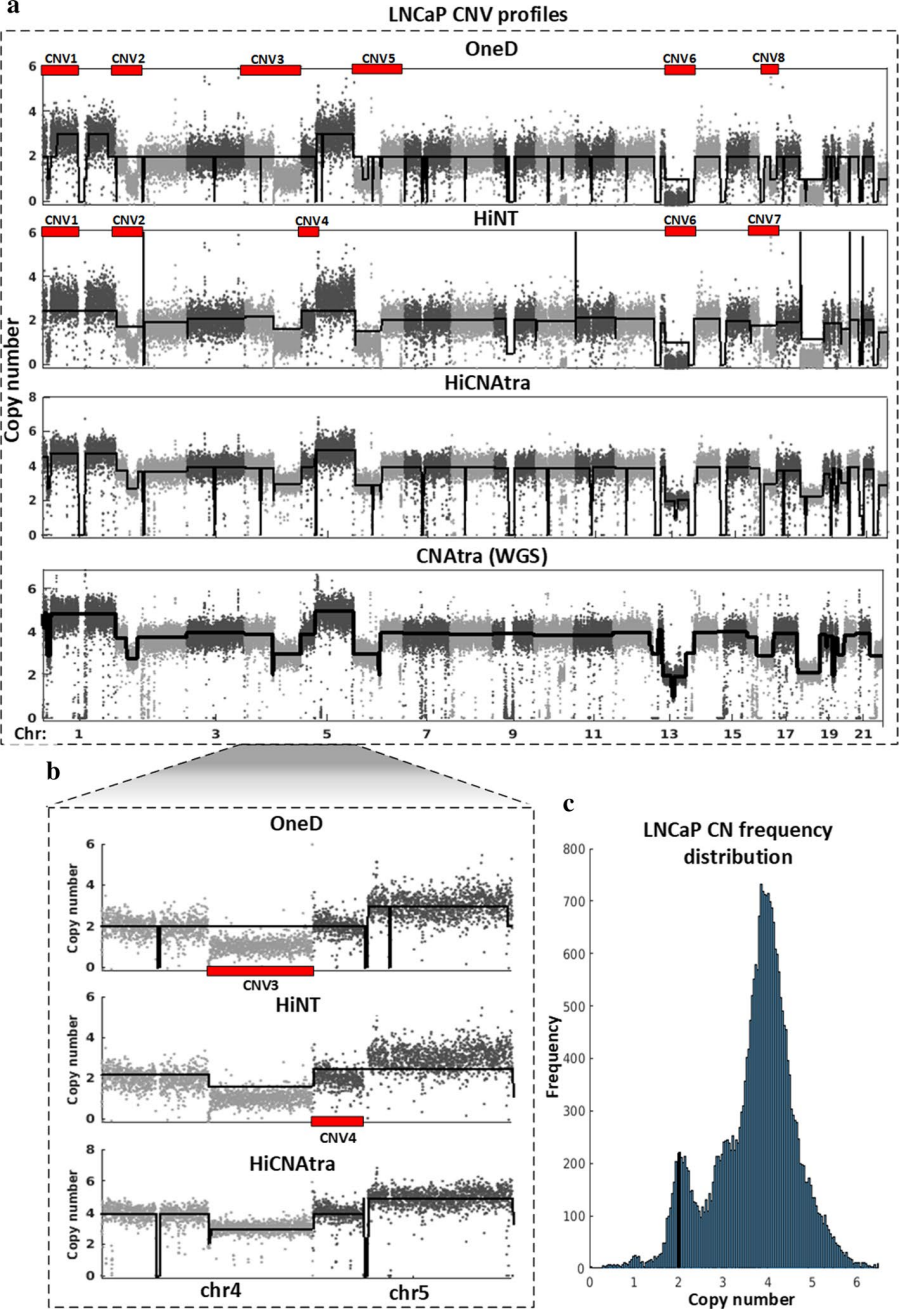
Visual comparison of the copy number profiles of LNCaP cancer cell line generated by OneD, HiNT and HiCNAtra. **a** Coverage plots showing genome-wide CNV profiles estimated by OneD (first panel), HiNT (second panel) and HiCNAtra (third panel) derived from LNCaP Hi-C data. The last panel shows the CNAtra estimated CNV profiles derived from WGS data of LNCaP. Note: The centromeres and telomeres of chromosomes are filtered out in CNAtra output. **b** Zoom-in view of the CNV profiles of chr 4 and 5 show that CNV3 and CNV4 are not detected by OneD and HiNT, respectively. Each grey dot represents the copy number of a bin. The black line represents the copy number track where any amplitude transition indicates a new CNV region. For HiCNAtra, the copy number track shown here is computed from the LCVs only. The red blocks indicate CNVs that are missed or incorrectly called by OneD or HiNT. **c** The CN frequency distribution exhibits the multimodality feature of the LNCaP cancer genome with most genomic segments having a copy number of 4. The vertical black line denotes the copy number reference (CN = 2)

Next, we benchmarked the HiCNAtra CNV caller module against HiNT as well as OneD’s CNV detection module using five Hi-C/3C-seq datasets of cancer cell lines (MCF7, LNCaP, PC3, K562, and H69AR). We applied HiNT on these datasets using interaction matrices binned at 50-kb (default bin size in HiNT) as well as 5-kb (default bin size in HiCNAtra) for a fair comparison. OneD uses 500-kb binning (heatmap resolution) for both CNV profiling and contact map correction. Our results demonstrated that HiNT and OneD failed to estimate the accurate copy number of genomic segments (Fig. 3, Additional file 1: Figs. S8–S12). For example, we generated the CNV profiles of chr 3 from Hi-C data of LNCaP, a hypotetraploid prostate cancer cell line with a modal chromosome number of 76–91 [39], using all three tools (Fig. 3a). Both HiNT and OneD failed to estimate the CN of most chromosomal regions of chr 3 and designated them as CN-neutral regions. In contrast, HiCNAtra correctly estimated the copy numbers of most ‘large’ segments in chr 3 as 4 N which corroborates well with hypotetraploid karyotype of LNCaP. More importantly, HiNT and OneD failed to detect a few visually-conspicuous CNVs, large regions with apparent amplitude-shift in the RD signal (Fig. 3a, CNV1–CNV8). In other cases, they merged genomic regions of different read counts into a single CN event (Fig. 3a, CNV1–CNV8). Similarly, OneD and HiNT could not detect the chr 4 CNV3 and chr 5 CNV4 regions, respectively (Fig. 3b). This may be attributed to the assumption of unimodal distribution and/or wrong estimation of CN reference (CN = 2). On the other hand, the multimodal model of HiCNAtra accurately fitted the copy number (normalized RD) frequency distribution of LNCaP cells (Fig. 3c) leading to accurate estimation of CN reference (CN = 2) and precise identification of change points of the RD signal as CN events (Fig. 3a, b). We witnessed similar observations on the CNV profiles of the MCF7 and PC3 cancer cell lines (Additional file 1: Fig. S8, S9). Further analysis of the CNV profiles derived from 3C-seq data of H69AR and K562 cell lines showed that OneD and HiNT failed to identify most of the CNV events (Additional file 1: Figs. S10, S11). In these examples, apart from the multimodality-associated problems, higher variability of RD signals was observed for these 3C-seq datasets. OneD and HiNT derive their RD signal from contact matrices represented by valid pairs only that constitute 13–15% of the total 3C-seq reads. This results in the generation of low-resolution RD signal, which adversely affects their performance in CNV detection. To further validate our conclusions, we visually examined the genome-wide RD signals that are generated as output files by the HiNT tool (Additional file 1: Fig. S12). We found that HiNT can correctly extract the RD signal from Hi-C datasets (MCF7 and LNCaP), but failed to capture the same in the case of 3C-seq data (H69AR). Taken together, the multimodal assumption of RD signal distribution is an essential key for accurate detection of CNVs in cancer cell lines. Additionally, in the case of 3C-seq data, it is imperative to utilize the genomic reads, which constitute the bulk of 3C-seq reads, to estimate the RD signal.

Finally, we analyzed the width of CNVs in MCF7, LNCaP, and K562 cancer cell lines estimated by HiNT with 5-kb and 50-kb binning, and OneD with 500-kb binning (Additional file 8: Table S7). We found that the median width of these CNVs ranges from 10.5 to 43.1 Mb, similar to the size range of LCVs detected by HiCNAtra, suggesting that both HiNT and OneD are optimized to detect large-scale CN alteration events but failed to detect sub-Mb-scale focal alterations. However, visual inspection of the CNV profiles generated from MCF7 Hi-C data (Additional file 1: Fig. S9) and H69AR 3C-seq data (Additional file 1: Fig. S11) clearly illustrated that chromosomes are interspersed with both large-scale and focal alteration events. In summary, HiCNAtra outperforms the OneD and HiNT tools in estimating the correct copy number as well as in detecting CNV events from Hi-C/3C-seq data.

### Explicit-based normalization method is better equipped to normalize CNV-driven bias on contact matrices of cancer cell lines

Several tools have been developed to remove the systematic biases and normalize the chromatin contact map using implicit- and explicit-based approaches. CNV is a hallmark of cancer cells. It is a well-established fact that the chromatin contact map is highly influenced by the CN change events [17, 18]. The copy number gains or losses result in apparent increment or decline in interaction frequencies creating visible artifacts on the chromatin contact map. This results in a fallacious interpretation of the higher-order chromatin conformations.

We, therefore, probed into the effects of CNV bias on chromatin contact map and evaluated the performance of HiCNAtra against different Hi-C correction tools in ameliorating their effects through visualization of pre- and post-corrected heatmaps and 1D signal variation. For performance evaluation, we compared HiCNAtra against the widely-used ICE method [27] as well as recently introduced, OneD [26] and CAIC [30] tools, that have been specifically developed to remove the CNV-driven bias from the contact map. It is important to note that CNV-targeted Hi-C correction tools, HiCNAtra, OneD, and CAIC, also remove other systematic biases (GC-content, effective fragment length, and mappability). On the other hand, the ICE method uses a matrix balancing approach to normalize all systematic biases with implicit assumptions and therefore expected to correct the bias introduced by CNVs as well. These four Hi-C correction tools can be mainly categorized into (1) implicit (ICE and CAIC) and (2) explicit-based methods (OneD and HiCNAtra). For this analysis, we used Hi-C data of the commonly-used breast cancer cell line MCF7 as these cells show abnormal karyotype and carry pervasive chromosomal alterations [40]. Besides, both OneD and CAIC tools used MCF7 for performance evaluation in their respective studies.

**Fig. 4:**
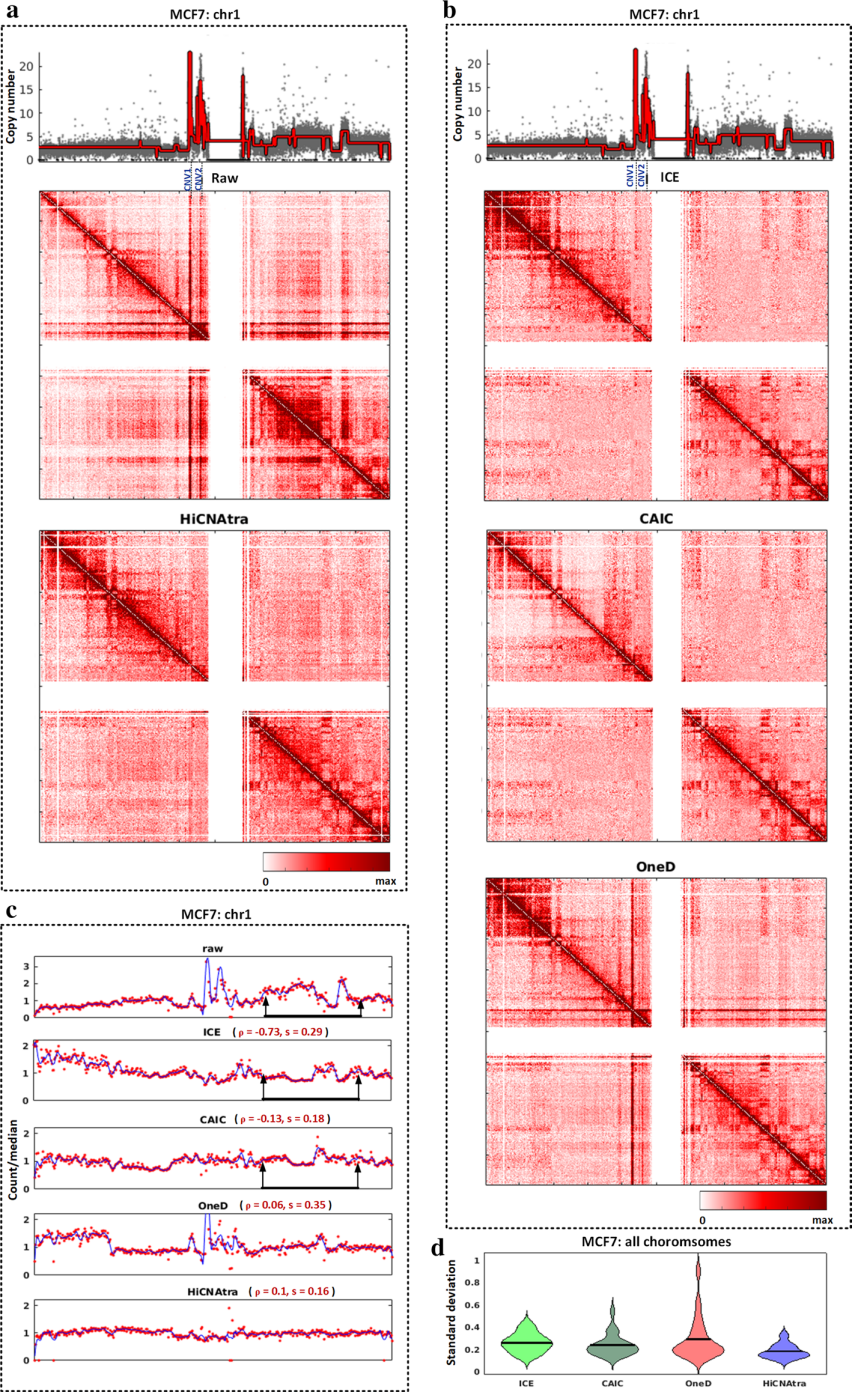
HiCNAtra normalization successfully ameliorates the effects of CNVs on the MCF7 chromatin contact map. **a** Raw (top) and HiCNAtra-normalized (bottom) interaction heatmaps (500-kb bin) of MCF7 chr 1. Copy number track of chr 1 computed by HiCNAtra from MCF7 Hi-C data is shown on top. Each grey dot represents the copy number of a bin. The red line represents the copy number track where any amplitude transition indicates a new CNV region. Two amplified regions, CNV1 and CNV2, are shown that resulted in two off-diagonal block patterns (visible artifacts). **b** Post-normalized Hi-C interaction heatmaps (500-kb bin) of MCF7 chr 1 using ICE (top), CAIC (middle), and OneD (bottom) tools. The copy number track is shown as in (**a**). For the OneD tool, *OneD* + *CN* normalization module was used. **c** Visual comparison of the 1D signals of pre-corrected (raw) and post-corrected contact maps of MCF7 chr 1 using different Hi-C correction tools. The five panels show the raw and post-corrected 1D signals (red dots) using ICE, CAIC, OneD, and HiCNAtra tools, respectively. The blue line shows the moving average of the 1D signal. The black line with arrows represents a region showing a signal inversion effect. For each post-corrected 1D signal, the Spearman correlation (ρ) and standard deviation (s) are indicated next to each panel. **d** Bean plot of standard deviations per chromosome of 1D signal of *cis* contact map corrected by ICE (green), CAIC (teal), OneD (red), and HiCNAtra (blue) tools

Visualization of contact heatmap is a simple and effective way to qualitatively ascertain the effects of CNVs on interaction frequencies. Therefore, we visually inspected the raw and corrected heatmaps generated by Hi-C correction tools (ICE, CAIC, OneD, and HiCNAtra) and evaluated their performance in ameliorating CNV-induced signal artifacts. Cancer cell lines, which typically carry widespread amplifications and deletions, create visible artifacts in the raw contact matrices (uncorrected heatmaps) (Fig. 4a, Additional file 1: Fig. S13). For instance, in MCF7 chr 1 heatmap, highly-amplified regions, CNV1 and CNV2, resulted in two off-diagonal block patterns with signal strength higher than their surrounding regions (Fig. 3a, shown by arrowhead). These blocks indicate the overrepresented *cis* interactions between these amplified regions with other genomic regions. As intended, HiCNAtra ameliorates CNV-induced artifacts as evidenced by the removal of off-diagonal blocks (artifacts) from the corrected heatmap without distorting the inherent chromatin conformation landscape, such as domain-like structural organization (Fig. 4a, Additional file 1: Fig. S13). Though the OneD-corrected heatmap showed improvement over the raw heatmap, the footprints of off-diagonal blocks of weaker strength are still visible in the post-corrected heatmap (Fig. 4b). We witnessed similar observations while inspecting other regions of MCF7 for OneD (Additional file 1: Fig. S13). In comparison, to the naked eye, the performances of iterative correction-based methods, ICE and CAIC, are on par with HiCNAtra in mitigating the CNV artifacts (Fig. 4b, Additional file 1: Fig. S13). This shows that HiCNAtra’s explicit approach is fairly adequate for correcting contact maps of cancer cell lines with abnormal karyotypes, similar to implicit approaches.

Nonetheless, visual inspection is not a gold standard to evaluate the performance of Hi-C correction tools. The main criteria for any normalization should be to attenuate the signal variations without changing the inherent behavior of the data. As defined by implicit-based approaches, the ultimate target of Hi-C normalization is to achieve near-equal visibility of all genomic regions. It is self-evident that this normalization must preserve the inherent behavior of the chromatin interaction signal. To evaluate the quality of normalization of Hi-C correction tools in attenuating the overall bias, we plotted 1D signal tracks (the summation of each column of the contact matrix) of raw and corrected heatmaps generated by ICE, CAIC, OneD, and HiCNAtra (Fig. 4c, Additional file 1: Fig. S14–S16, Additional file 9: Table S8). Visually, the HiCNAtra-corrected 1D tracks showed the minimum overall variation compared to the other Hi-C correction tools (Fig. 4c, Additional file 1: Fig. S14–S16). This is further substantiated by the fact that HiCNAtra achieved the least standard deviation in the 1D signal for all chromosomes (Fig. 4d). All other tools attenuate the 1D signal variation compared to the raw (uncorrected) signal albeit to varying degrees (Additional file 9: Table S8). The iterative-correction-based methods, CAIC and ICE, achieved the second-least and third-least variation levels, respectively (Fig. 4c).

Intriguingly, upon close inspection, we observed the ‘flipping’ (signal inversion effect) of the signal in ICE-corrected 1D trajectory relative to the raw 1D signal (Fig. 4c and Additional file 1: Fig. S14, shown by a black line). This observation is further supported by the fact that the ICE-corrected 1D signal of all MCF7 chromosomes showed negative correlations compared to the raw 1D signal (Additional file 10: Table S9). This signal inversion effect contradicts the general idea of normalization which tends to attenuate the bias effect without distorting the main feature of the signal. This also confirms the conclusions of previous studies that ICE results in overcompensation of original CNV bias leading to distortion of the interaction frequency signal, which render it unsuitable for correcting Hi-C contact map of cancer cells [26, 30]. Though CNV-targeted CAIC tool attempted to overcome this problem by utilizing CNV information in their matrix correction, its corrected 1D signal still carries the footprints of signal inversion effect (Fig. 4c and Additional file 1: Fig. S14, shown by a black line) as evidenced by the negative correlation of their 1D signal relative to the uncorrected ones in 16 chromosomes (Additional file 10: Table S9). It is notable that CAIC also utilizes local iterative correction in its approach. This data suggests that the iterative correction-based approach may not be suitable for normalizing Hi-C data of CNV-infested cancer cells. Additionally, signal inversion effects witnessed in implicit-based normalization approaches render the explicit-based Hi-C contact map correction as a better alternative for removing CNV bias.

### HiCNAtra is the best explicit-based method for correcting chromatin contact map from all systematic biases

After establishing the advantage of explicit-based approaches in normalizing CNV-driven bias, we comprehensively evaluated the performance of the two explicit-based Hi-C correction tools, HiCNAtra and OneD, for correcting all systematic bias sources (GC-content, mappability, effective fragment length, and CNV) using five Hi-C/3C-seq datasets of cancer cell lines (MCF7, LNCaP, PC3, H69AR, and K562). Although both tools utilize GLMs based on either Poisson (HiCNAtra) or negative binomial (NB) (OneD) distribution for contact map normalization, there are two major differences between OneD and HiCNAtra approaches. First, OneD uses an HMM-based CNV calling method, whereas HiCNAtra employs a multimodal-based hierarchical CNV calling approach. Second, OneD uses a two-step normalization approach while HiCNAtra simultaneously normalizes the contact map from all biases, including CNV-driven bias using a single model.

**Fig. 5:**
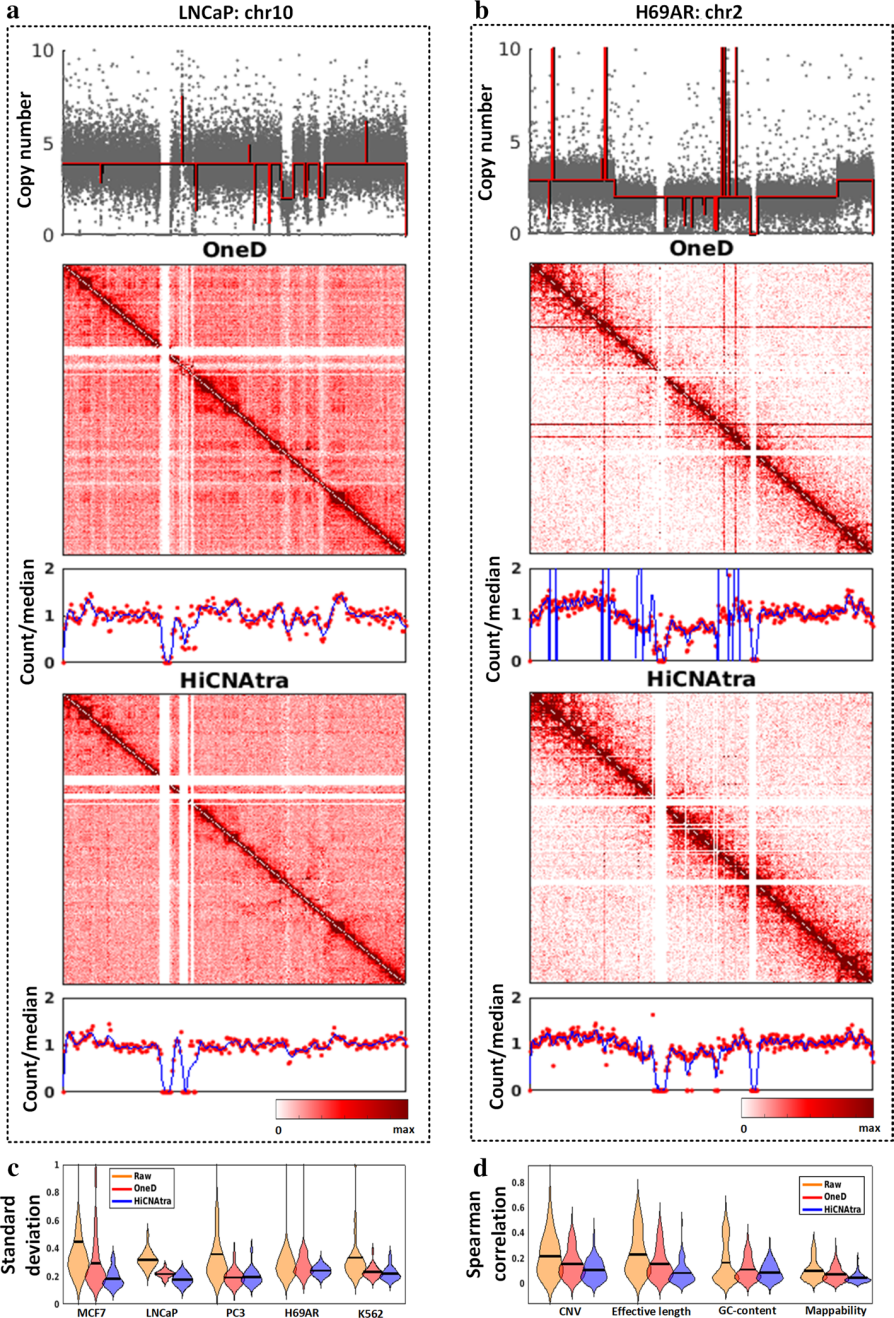
HiCNAtra removes sample-dependent and sample-independent biases from the contact maps of cancer cell lines. **a-b** Interaction heatmaps (500-kb bin) and 1D signals of LNCaP chr 10 (**a**) and H69AR chr 2 (**b**) corrected by OneD and HiCNAtra approaches. For the OneD tool, *OneD* + *CN* normalization module was used in all the analyses. Copy number track computed by HiCNAtra is shown on top. Each grey dot represents the copy number of a bin. The red line represents the copy number track where any amplitude transition indicates a new CNV region. The second and fourth panels show the OneD-corrected, and HiCNAtra-corrected contact maps, respectively. The third and fifth panels show post-corrected 1D signals (red dots) using OneD and HiCNAtra tools, respectively. The blue line shows the moving average of the 1D signal. **c** Bean plot of standard deviations per chromosome of 1D signal of raw (orange), OneD-corrected (red) and HiCNAtra-corrected (blue) *cis* contact maps computed from five cancer datasets (MCF7, LNCaP, PC3, H69AR, and K562). **d** Bean plot showing Spearman correlations between *cis* contact frequencies and sample-dependent (CNV) and sample-independent (effective length, GC-content, and mappability) biases in raw (orange), OneD-corrected (red) and HiCNAtra-corrected (blue) contact maps. Correlations are calculated across chromosomes of five cancer datasets

At the outset, we visually inspected the interaction heatmap (raw, OneD-corrected, and HiCNAtra-corrected) and 1D signal track of chromosomes carrying CNVs in different cancer cell lines (LNCaP chr 10, H69AR chr 2, PC3 chr 14, and K562 chr 3) (Fig. 5a, b and Additional file 1: Fig. S17). In all chromosomes, HiCNAtra successfully ameliorated the CNV-induced artifacts, as evidenced by the homogeneous signal in the corrected contact map, without any distortion of the inherent chromatin interaction landscape. In contrast, the footprints of these artifacts are conspicuous in the OneD-corrected heatmaps. Additionally, the 1D signals of HiCNAtra-corrected chromosome-wide heatmaps showed lesser variations compared to OneD (Fig. 5a, b and Additional file 1: Fig. S17). This is further confirmed by the fact that HiCNAtra scored the least standard deviation of genome-wide 1D signal variation in all cell lines (Fig. 5c, Additional file 11: Table S10).

Both OneD and HiCNAtra tend to explicitly estimate and correct the impact of all systematic bias sources (GC-content, mappability, effective fragment length, and CNV) on the interaction matrices. It is targeted to produce corrected heatmaps that are uncorrelated with these systematic biases. Therefore, we next measured the Spearman correlations between different biases and the interaction frequencies of the raw (uncorrected), OneD-corrected, and HiCNAtra-corrected contact maps. Our analysis of the five cancer cell lines demonstrated that raw interaction frequencies are positively correlated with the HiCNAtra-derived CNV track derived from Hi-C/3C-seq data of the same cell line (Fig. 5d and Additional file 12: Table S11), which reiterates the adverse impact of CNV on contact map as reported earlier [26, 30]. We found that CNV and effective fragment length impart the dominant biases on the raw contact map of cancer cell lines (Fig. 5d and Additional file 12: Table S11). For example, the Spearman correlation of genome-wide interaction frequencies with CNV is 0.47 for MCF7 and 0.51 for LNCaP, whereas Spearman correlation with effective fragment length is 0.34 for MCF7 and 0.41 for LNCaP. We observed that HiCNAtra-corrected interaction frequencies became virtually uncorrelated as they achieved the minimum correlation with CNV tracks as well as with other systematic biases across cell lines. For example, post-correction Spearman correlation of MCF7 genome-wide interaction frequencies with CNV is 0.1 for HiCNAtra and 0.28 for OneD. Similarly, for LNCaP, the post-correction Spearman correlation is 0.134 for HiCNAtra and 0.29 for OneD. Collectively, these data suggest that the HiCNAtra approach can successfully diminish the effects of CNV and other systematic biases.

It is intriguing that OneD has not performed on par with HiCNAtra although both tools are based on an explicit-based approach. Therefore, the sub-optimal performance of OneD can be attributed to either its CNV calling approach and/or its *sequential* bias correction method. We earlier demonstrated that HiCNAtra’s CNV caller module outperforms the CNV detection method of OneD (Fig. 3). To further probe into this aspect, we implemented a ‘*modified OneD*’ method where we incorporated HiCNAtra-generated CNV tracks as input in the original OneD tool instead of their default HMM-based CNV information for contact map normalization. We applied this *modified OneD* method to the five Hi-C/3C-seq datasets of cancer cell lines. We observed that *modified OneD*-normalized heatmap still harbors the footprint of off-diagonal blocks (artifacts) of weaker strength (Additional file 1: Figs. S18, S19). Nevertheless, the *modified OneD-*corrected heatmap showed less variation in the 1D signal compared to the original (default) OneD method (Additional file 1: Fig. S18, S19, Additional file 11: Table S10), suggesting that the utilization of HiCNAtra’s CNV information alone enhances the performance of *modified OneD*. This underscores the cardinal importance of accurate estimation of CNV profiles from Hi-C/3C-seq data for contact map normalization of cancer cell lines with pervasive chromosomal aberrations. Strikingly, the HiCNAtra-corrected interaction frequencies still showed the minimum overall variations in the 1D signal compared to *modified OneD* (Additional file 1: Figs. S18, S19, S20a, Additional file 11: Table S10). Furthermore, HiCNAtra achieved the minimum correlations with CNV tracks as well as with other systematic biases across cell lines (Additional file 1: Fig. S20b and Additional file 12: Table S11). For example, Spearman correlation of genome-wide interaction frequencies with CNV is 0.1 (MCF7) and 0.13 (LNCaP) for HiCNAtra and 0.24 (MCF7) and 0.33 (LNCaP) for *modified OneD*.

Taking together, we speculate that the advantage of HiCNAtra’s normalization method is attributed to (1) the improved RD calculation from Hi-C/3C-seq reads, (2) the multimodal-based hierarchical CNV calling approach, and (3) the *simultaneous* bias correction of Hi-C contact map.

## Discussion

Cancer genomes are scattered with large-scale and focal CNVs. Amplification and deletion events will naturally result in over- and under-representation of chromatin interactions respectively in Hi-C/3C-seq contact matrices. As previously reported [18, 26, 30], our analysis also pointed out a positive correlation between the copy number and the strength of the interaction frequencies of genomic loci. This clearly suggests that CNV presents a dominant explicit bias in the case of cancer cell lines with abnormal karyotypes. Therefore, CNVs can greatly influence the interpretation of chromatin contact maps and may lead to false identification of genomic regions with over- or under-represented interaction strengths. The effect of CNV-driven bias should be compensated for the amplified/deleted regions along with other systematic biases for accurate interpretation of interaction frequency with respect to the CN-neutral regions. Therefore, we have improved on three aspects for the effective normalization of chromatin contact map of cancer cell lines.

High-resolution extraction of RD signal from Hi-C/3C-seq datasets is central to discover copy number events of smaller sizes. We proposed two solutions to better estimate the RD signal from Hi-C datasets. First, HiCNAtra utilizes all types of Hi-C reads, resulting in high-coverage RD signal, unlike other tools that use only valid pairs. Second, HiCNAtra employs a novel entire-fragment counting approach to create the RD signal track that is consistent with the WGS-derived RD profile of the corresponding cell line.

Accurate estimation of the CNV profile is a prerequisite for effectual normalization of the chromatin contact map. To achieve this, we adapted multimodal distribution-based modeling of RD signal for correct estimation of CN reference (CN = 2), which allows hierarchical identification of large-scale (Mb-scale) and focal alterations (kb-scale) in cancer cell lines with hyperploid karyotypes.

For normalization, HiCNAtra utilizes a Poisson regression model to learn the effects of CNVs on the contact map in conjunction with other systematic biases. Therefore, HiCNAtra is able to estimate the contributions of each bias simultaneously instead of merely assuming a pre-defined specific effect of the CNVs on the interaction frequencies. HiCNAtra thus employs a GLM to normalize the contact frequencies (the variable) versus GC-content, effective fragment length, mappability and CNV (the predictors) simultaneously. In principle, HiCNAtra can also include newer sources of biases in the same method. Therefore, it is adaptable to be future-ready for correcting new biases from chromatin interaction data. HiCNAtra alleviates the CNV-induced visual artifacts from raw contact maps as evidenced by near-uncorrelation of contact frequencies with the CNV regions.

In this study, our benchmarking analysis of the HiCNAtra’s CNV detection module against OneD and HiNT firmly established the advantage of implementing multimodal-based CNV discovery. Similarly, RD signal extraction using the entire-fragment counting approach for Hi-C reads facilitated the detection of focal copy number alterations. Performance evaluation of contact map correction against ICE, OneD, and CAIC methods undeniably demonstrated that HiCNAtra is superior to other Hi-C correction tools in eliminating the CNV effects on contact matrices without any signal inversion effect.

It is worth mentioning that previous studies have demonstrated the adverse effects of CNVs on the higher-order chromatin structure such as TADs [17, 18]. However, chromatin topological structures such as TADs and A/B compartments are defined by computational methods using a normalized Hi-C contact map. As we have shown in our data, the contact map exhibits signal artifacts in CNV regions. Hence, whether the TAD changes demonstrated in these studies are real alterations in higher-order chromatin organization or a result of CNV artifacts on the Hi-C contact map is open to interpretation. We believe that an in-depth study supported by sound experimental validation is necessary to provide the true picture of chromatin organization in cancer cells.

Altogether, our results suggest that HiCNAtra provides a better solution for (a) computing high-coverage RD signal and detecting large-scale and focal CNVs from Hi-C/3C-seq datasets, and (b) for explicitly correcting the chromatin contact frequencies from all systematic biases, including bias introduced by CNVs, in aneuploid cancer cell lines.

## Conclusions

HiCNAtra is a computational framework for CNV detection from Hi-C data and systematic bias removal from chromatin contact matrices. For this, HiCNAtra employs a three-pronged approach. First, using the entire-fragment counting approach, it extracts a high-resolution RD signal utilizing all Hi-C reads. Second, a multimodal-based CNV detection allows accurate estimation of CNV profiles of cancer cell lines. Finally, HiCNAtra’s explicit normalization provides the best solution for contact map correction of cancer cell lines by simultaneously integrating all the systematic biases. To sum up, HiCNAtra is the best tool available to date for detecting CNVs from Hi-C/3C-seq data as well as for correcting chromatin contact frequencies.

## Methods

Here, we explain the main modules of the HiCNAtra framework. The description of the wet lab experiments and other modules of the HiCNAtra pipeline are provided in the Extended Methods under Additional file 1. The HiCNAtra software is freely available along with the user manual that contains all the necessary information about the input data and all output results. This software also provides an interactive platform to visualize and manually inspect the complete CNV profiles, pre- and post-normalized contact maps, and accessory information for further validation and interpretation.

### HiCNAtra framework

HiCNAtra pipeline is divided into three modules (Fig. 1a): (1) computation of the RD signal from Hi-C or 3C-seq reads (*RD calculator*), (2) RD-based detection of copy number events (*CNV caller*), and (3) bias correction of interaction matrix (*contact map normalization*). Briefly, the HiCNAtra pipeline starts with computing RD signal from the Hi-C/3C-seq reads. Next, we employ CNAtra [23] approach to identify large-scale and focal alterations from the RD signal and integrate them to generate the CNV track that is used as an explicit bias source for correcting chromatin interaction matrix. Finally, we utilize Poisson-based GLM to simultaneously normalize the contact map for GC-content, mappability, and effective fragment length biases as well as bias introduced by copy number gains/losses.

#### Computing the RD signal from Hi-C/3C-seq NGS reads

Hi-C/3C-seq datasets comprise different read types such as valid pairs, dangling-end, extra dangling-end, self-circle, and single-sided reads as well as genomic reads [41]. These reads can be mainly categorized into (1) ‘informative’ reads containing valid pairs that represent interactions between genomic loci, and (2) ‘non-informative’ reads that include all other types of reads. In Hi-C datasets analyzed in this study, valid pairs comprise 39–64% of the total mapped reads (Additional file 2: Table S1). These valid pair reads are solely used to generate the contact map. HiCNAtra integrates other non-informative reads (dangling-end, extra dangling-end, self-circle, and single-sided reads) along with valid pairs to compute the RD signal at a higher resolution. On the other hand, in the absence of biotin labeling and pull-down steps, the majority of 3C-seq reads are contributed by genomic reads. Therefore, for 3C-seq datasets, we compute the RD signal exclusively from these genomic reads similar to RD signal computation from paired-end WGS reads. Depending on the Hi-C or 3C-seq experiment, reads are utilized differently for computing the RD signal (default bin size = 5 kb).

For Hi-C datasets, HiCNAtra computes the RD signal from all Hi-C reads (Fig. 1b, top panel) using the entire-fragment counting approach. For this, we first retain only those reads that are located within the restriction fragment-end windows – a region of maximum molecule length adjacent to the restriction site (Fig. 1b, second panel). Second, we count the reads for each restriction fragment based on the assumption that each continuous DNA-sequence read represents a particular restriction fragment and contributes to the abundance of that fragment. Therefore, we count each non-informative read as a single count, whereas for valid pairs, side1 and side2 are counted separately (Fig. 1b, third panel). So, for a particular restriction fragment, the fragment count is calculated as the sum of the number of reads located in both fragment-end windows (Fig. 1b, bottom panel). For example, the fragment count of restriction fragment 1 (rFrag1) is the sum of read counts of fragment-end windows, w2 and w3 (Fig. 1b, bottom panel). Finally, we assign the fragment count of a restriction fragment to all its bases and compute the RD signal. Then, we corrected it against all systematic biases (GC-content, mappability, and effective length features) (See Additional file 1). Differential restriction fragment lengths can affect the coverage of a particular restriction fragment in the Hi-C data [24]. Effective length feature is defined as the number of nucleotides belongs to the restriction fragment-end windows per bin.

For 3C-seq datasets, genomic reads are distributed uniformly along the genome and represent the majority of reads (Fig. 1b, top panel). Therefore, we use these paired-end genomic reads (Fig. 1b, middle panel) to compute the RD signal in an unbiased manner (Fig. 1b, bottom panel).

#### CNV identification

The CNV caller module of HiCNAtra is based on CNAtra [23] approach. CNAtra framework constitutes the hierarchical framework to delineate the multi-level copy number alterations in the cancer genomes. Briefly, the CN reference (CN = 2) is first defined by fitting the RD signal to a multimodal distribution. Then, a multi-step framework is utilized to initially identify large genomic segments with distinct CN states. Segments with CN state other than 2 (CN ≠ 2) are considered as LCVs or segmental aneuploidies. Next, FAs containing focal amplifications and deletions in each CN-defined segment are detected based on coverage-based thresholding. HiCNAtra finally computes the CNV track by merging both LCVs and FAs for Hi-C correction.

#### Correction of the chromatin contact map

We employed a GLM to normalize the contact frequencies against all sources of biases. This GLM model was first introduced by HiCNorm [32] to correct systematic biases introduced by restriction fragment length, mappability and GC-content, excluding CNV bias. In the HiCNorm study, the comparative analysis showed that both Poisson and NB distributions performed equally in bias reduction. Nevertheless, HiCNorm uses the Poisson distribution model as it is mathematically simpler. Two GLMs with Poisson distribution are used to fit the *cis* and *trans* contact maps separately. By default, we apply our normalization approach in a genome-wide manner for better estimation of the GLM parameters.

#### Normalization of cis contact map

Let $\user2{ }F = \left\{ {f_{kl}^{i} } \right\}_{{1 \le k,l \le n_{i} }}$ represent the $n_{i} \times n_{i}$ *cis* interaction frequencies for chromosome $i$, where $n_{i}$ is the number of bins in chromosome $i$. Each element $f_{kl}^{i}$ represent the number of interactions between genome loci from bin $k$ and bin $l$ in chromosome $i$. Let $x_{k}^{i}$, $y_{k}^{i}$, $z_{k}^{i}$, and $w_{k}^{i}$ represent the GC content, mappability, effective fragment length, CNV features of bin $k$ in chromosome $i$, respectively; whereas $x_{l}^{i}$, $y_{l}^{i}$, $z_{l}^{i}$, and $w_{l}^{i}$ represent the features of bin $l$ in chromosome $i$, respectively. We assume that the interaction frequency $f_{kl}^{i}$ follows a Poisson distribution with rate $\lambda_{jk}$:

$$\log \left( {\lambda_{jk} } \right) = \alpha_{0}^{i} + \alpha_{1}^{i} *\log \left( {x_{k}^{i} x_{l}^{i} } \right) + \alpha_{2}^{i} *\log \left( {y_{k}^{i} y_{l}^{i} } \right) + \alpha_{3}^{i} *\log \left( {z_{k}^{i} z_{l}^{i} } \right) + \alpha_{4}^{i} *\log \left( {w_{k}^{i} w_{l}^{i} } \right),$$

where $\alpha^{i}_{0 - 4}$ are the coefficients of the GLM model. We fit this GLM model and use the coefficient estimates $\hat{\alpha }_{0 - 4}$ for computing the estimated Poisson rates:

$$\hat{\lambda }_{kl} = {\exp}\left[ {\hat{\alpha }_{0}^{i} + \hat{\alpha }_{1}^{i} *\log \left( {x_{k}^{i} x_{l}^{i} } \right) + \hat{\alpha }_{2}^{i} *\log \left( {y_{k}^{i} y_{l}^{i} } \right) + \hat{\alpha }_{3}^{i} *\log \left( {z_{k}^{i} z_{l}^{i} } \right) + \hat{\alpha }_{4}^{i} *\log \left( {w_{k}^{i} w_{l}^{i} } \right)} \right].$$

Then, $\hat{\lambda }_{kl}$ is used to compute the normalized interaction frequency $\hat{f}_{kl}^{i}$ between bin $k$ and bin $l$ in chromosome $i$ of bin as follows:

$$\hat{f}_{kl}^{i} = \frac{{f_{kl}^{i} }}{{\hat{\lambda }_{kl} }}$$

#### Normalization of trans contact map

Let $F = \left\{ {f_{kl}^{ij} } \right\}_{{1 \le k \le n_{i} , 1 \le l \le n_{j} }}$ represent the $n_{i} \times n_{j}$ *trans* interaction frequencies between chromosome $i$ and chromosome $j$, where $n_{i}$ and $n_{j}$ are the number of bins in chromosomes $i$ and $j$, respectively. Each element $f_{kl}^{ij}$ represent the number of interactions between genome loci of bins $k$ and $l$ from chromosomes $i$ and $j$, respectively. Let $x_{k}^{i}$, $y_{k}^{i}$, $z_{k}^{i}$, and $w_{k}^{i}$ represent the GC content, mappability, effective fragment length, CNV features of bin $k$ in chromosome $i$, respectively; whereas $x_{l}^{j}$, $y_{l}^{j}$, $z_{l}^{j}$, and $w_{l}^{j}$ represent the features of bin $l$ in chromosome $j$, respectively. We assume that the interaction frequency $f_{kl}^{ij}$ follows a Poisson distribution with rate $\lambda_{jk}$:

$$\log \left( {\lambda_{jk} } \right) = \alpha_{0}^{ij} + \alpha_{1}^{ij} *\log \left( {x_{k}^{i} x_{l}^{j} } \right) + \alpha_{2}^{ij} *\log \left( {y_{k}^{i} y_{l}^{j} } \right) + \alpha_{3}^{ij} *\log \left( {z_{k}^{i} z_{l}^{j} } \right) + \alpha_{4}^{ij} *\log \left( {w_{k}^{i} w_{l}^{j} } \right),$$

where $\alpha^{ij}_{0 - 4}$ are the coefficients of the GLM. We fit this GLM model and use the coefficient estimates $\hat{\alpha }_{0 - 4}$ for computing the estimated Poisson rates:

$$\hat{\lambda }_{kl} = {\exp}\left[ {\hat{\alpha }_{0}^{ij} + \hat{\alpha }_{1}^{ij} *\log \left( {x_{k}^{i} x_{l}^{j} } \right) + \hat{\alpha }_{2}^{ij} *\log \left( {y_{k}^{i} y_{l}^{j} } \right) + \hat{\alpha }_{3}^{ij} *\log \left( {z_{k}^{i} z_{l}^{j} } \right) + \hat{\alpha }_{4}^{ij} *\log \left( {w_{k}^{i} w_{l}^{j} } \right)} \right].$$

Then, $\hat{\lambda }_{kl}$ is used to compute the normalized interaction frequency $\hat{f}_{kl}^{ij}$ between bins $k$ and $l$ from chromosomes $i$ and $j$, respectively:

$$\hat{f}_{kl}^{ij} = \frac{{f_{kl}^{ij} }}{{\hat{\lambda }_{kl} }}.$$

### Data processing

We used six publicly available Hi-C datasets (GM12878, IMR90, PrEC, MCF7, LNCaP, and PC3) and two in-house generated 3C-seq datasets (K562 and H69AR). We used input control reads of ChIP-seq experiments of MCF7, LNCaP, and K562 cells to generate WGS-derived RD signal to validate HiCNAtra-computed RD signal. Detailed information about the sequencing datasets is provided in Additional file 2: Table S1. The wet lab experimental details about cell culture and 3C-seq library preparation are provided in Extended Methods under Additional file 1.

The iterative mapping has been shown to recover the maximum numbers of aligned reads compared to fixed-length mapping [27]. Therefore, for both Hi-C and 3C-seq datasets, we applied the iterative-mapping technique of hiclib using default parameters for aligning short sequence reads to human reference genome GRCh37 (hg19). The iterative mapping output HDF5 files are used as input for the HiCNAtra tool. Classification of read types (valid pairs, dangling-end, extra dangling-end, self-circle) depends on the maximum molecule length (MML). For each dataset, we approximately set the MML as the size (in hundreds of bp) that is greater than the fragment lengths (side1-start to side2-end) of 99% of dangling-end and extra-dangling end reads (Additional file 2: Table S1, Additional file 1: Fig. S21).

## Supplementary information

**Additional file 1.** Supplementary Information containing Extended Methods and Supplementary Figures.

**Additional file 2**. Supplementary Table S1.

**Additional file 3**. Supplementary Table S2.

**Additional file 4**. Supplementary Table S3.

**Additional file 5**. Supplementary Table S4.

**Additional file 6**. Supplementary Table S5.

**Additional file 7**. Supplementary Table S6.

**Additional file 8**. Supplementary Table S7.

**Additional file 9**. Supplementary Table S8.

**Additional file 10**. Supplementary Table S9.

**Additional file 11**. Supplementary Table S10.

**Additional file 12**. Supplementary Table S11.

## Data Availability

Supplementary Information containing Extended Methods and Supplementary Figures as well as Supplementary Tables are provided as Additional files. All the datasets used in this study are publicly available (Additional file 2: Table S1). The 3C-seq datasets reported in this study are available through NCBI BioProject accession number PRJNA658851.

## Abbreviations

3C: Chromosome conformation capture
3D: Three-dimensional
CAIC: CNV-adjusted iterative correction
chr: Chromosome
CN: Copy number
CNV: Copy number variation
CS: Centralization score
FA: Focal alteration
GAM: Generalized additive model
GLM: Generalized linear model
HMM: Hidden Markov model
ICE: Iterative correction and eigenvector decomposition
LCV: Large-scale copy number variation
NGS: Next-generation sequencing
NB: Negative binomial
MML: Maximum molecule length
RD: Read depth
TAD: Topologically-associating domain
WGS: Whole-genome sequencing
ZS: Zero score
