# Supplementary material for: Identification and utilization of copy number information for correcting Hi-C contact map of cancer cell lines
Source: BMC Bioinformatics. 2020 Nov 7;21:506. doi: 10.1186/s12859-020-03832-8 (PMC7648276; doi:10.1186/s12859-020-03832-8)
Supplement: Supplementary file 1 — Additional file 1. Supplementary Information containing Extended Methods and Supplementary Figures. [file 12859_2020_3832_MOESM1_ESM.pdf]

Supplementary Information:

Identification and Utilization of Copy Number Information for Correcting Hi-C Contact Map of Cancer Cell Lines

Ahmed Ibrahim Samir Khalil[1], Siti Rawaidah Binte Mohammad Muzaki[2], Anupam Chattopadhyay[1,*], Amartya Sanyal[2,*]

[1]School of Computer Science and Engineering, Nanyang Technological University, 50 Nanyang Avenue, Singapore 639798.
[2]School of Biological Sciences, Nanyang Technological University, 60 Nanyang Drive, Singapore 637551.

[*]Corresponding e-mail: anupam@ntu.edu.sg; asanyal@ntu.edu.sg

EXTENDED METHODS

1. Cell line and cell culture

Human chronic myelogenous leukemia cell line K562 was cultured in RPMI 1640 medium with 10% fetal bovine serum and supplemented with 1X Penicillin-Streptomycin solution (HyClone, Cat# SV30010) and 2mM L-glutamine (HyClone, Cat# SH30034.01). The multidrug-resistant cell line, H69AR (ATCC CRL-11351), was cultured in RPMI 1640 growth medium as mentioned above with 20% fetal bovine serum. H69AR cells were cultured in presence of 0.8 μM of doxorubicin (Fisher Scientific, Cat# BP251610) once a month for 3 days to maintain the doxorubicin resistance. Both cell lines were cultured at 37°C in a humidified CO_2 incubator.

2. 3C-seq library preparation

The 3C library for H69AR was prepared using 2 x 10^7 cells according to the published protocol [1-3] with some modifications. Cells were chemically crosslinked by adding formaldehyde (1% final concentration) and incubated at room temperature for 10 min. The crosslinking reaction was terminated by adding glycine at a final concentration of 125mM and incubated at room temperature for 15 minutes. Crosslinked cells were pelleted by centrifugation and the cell pellet was stored at -80°C till further use. For H69AR 3C library preparation, crosslinked cells were thawed on ice and resuspended in ice-cold lysis buffer (10 mM Tris-HCl pH 8.0, 10 mM sodium chloride, 0.2% (v/v) Igepal CA-630 plus protease inhibitor cocktail added before use) and the cell suspension was incubated for at least 15 min. The cells were homogenized by stroking 20 times with a large-clearance Dounce homogenizer. The suspension was centrifuged and the pellet was collected and washed with chilled 1X DpnII buffer (NEB, Cat#B7006S). After centrifugation, the pellet was collected and resuspended in 250 μl 1X DpnII buffer. The suspension was distributed into five microfuge tubes and the volume of each was made up to 362 μl with 1X DpnII buffer. In each tube, chromatin was solubilized by adding 38 μl of 1% SDS and incubating the tube at 65°C for exactly 10 min. The SDS was quenched by adding 44 μl of 10% Triton X-100 and the chromatin was digested overnight at 37°C with 1000 units of DpnII (NEB, Cat# R0543M). The restriction enzyme was heat-inactivated at 65°C for 30 min and the digested chromatin was transferred to a 15-ml tube containing ice-cold 7.61 ml ligation master mix (745 μl 10X ligation buffer, 80 μl 10 mg/ml BSA, 80 μl 100 mM ATP and 6705 μl water). The ligation reaction was carried out by adding 50 units of T4 DNA ligase (1 U/μl) (ThermoFisher Scientific, Cat#15224090) and incubated for 4 hours at room temperature. To

reverse the crosslinking, 100 µl of 10 mg/ml Proteinase K (ThermoFisher Scientific Cat #25530031) was added to the tube and incubated overnight at 65°C. Following this, DNA was purified using the standard phenol-chloroform extraction procedure. After ethanol precipitation, the DNA was resuspended in 500 µl of 1X Tris-EDTA (TE) buffer pH 8.0. The DNA solution was subsequently desalted using a 0.5 ml Amicon Ultra centrifugal filter (30K column) (Merck, Cat#C82301). Desalting was accomplished by concentrating the sample and reconstituting the original sample to 0.5 ml with 1X TE buffer. This step was repeated 3 times to remove any traces of salt. Purified DNA was collected by inverting the column into a clean collection tube by centrifuging at 1,000g for 2 min and the total volume was made up to 100 µl with 1X TE buffer. The RNA was degraded by adding 1 µl of 10 mg/ml RNase A and incubated for 15 min at 37°C and the DNA containing 3C library was stored at -20°C. For Illumina NGS library preparation, the 3C library was transferred into a Covaris AFA microtube and sonicated using a Covaris ultrasonicator to a size range of 100-1000 bp. AMPure XP bead-based size selection was then carried out to obtain DNA fragments of 300-600 bp size range. Illumina paired-end NGS library for H69AR was prepared using ThruPLEX DNA-Seq Kit (Takara Bio). For K562 cells, 2 x 10^6 cells were used for preparing the 3C-seq library using DpnII enzyme. A rapid protocol standardized in the lab, which utilizes low reaction volume and low-input sample, was used to generate K562 3C library. The paired-end NGS library was prepared using an in-house standardized protocol. Both H69AR and K562 3C-seq libraries were sequenced on an Illumina HiSeq2500 (2 x 101 bp) platform by an external service provider.

3. HiCNAtra pipeline

3.1 Extracting the bias features of RD signal

After computing the RD signal, we correct it from all systematic biases (mappability, GC-content, and effective fragment length). For that, we used the unique mappability track [4], Miller's GC-track [5] and the reference human genome sequence. For 3C-seq data, GC-content and mappability score per bin are computed using CNAtra approach [6]. For Hi-C data, GC-content and mappability score per bin are computed based on entire-fragment counting approach (Fig. 1b). In addition, effective length feature is calculated as the number of nucleotides belongs to fragment-end windows per bin.

3.2 Normalization of the RD signal

RD signal is first filtered to remove the low-mappability regions, gap regions and ENCODE blacklisted regions of the human genome (described in [6]). Then, we normalize the RD signal for the systematic biases by employing interval normalization method [6, 7]. For each bias source, the interval of bias values is divided into 100 equally spaced sub-intervals. A correction factor is computed for each sub-interval as the ratio between the genome-wide mean of the RD signal to the average RD signal of bins with bias value in this sub-interval. Then, the correction factors are used for computing the normalized RD signal.

$$\mathrm{RD}_{\mathrm{corrected}}^{\mathrm{i,j,k}} = \mathrm{RD}_{\mathrm{raw}}^{\mathrm{i,j,k}} \times G^{i} \times M^{j} \times E^{k}$$

$$G^{i} = \frac{\overline{\mathrm{RD}}_{\mathrm{genome}}}{\overline{\mathrm{RD}}_{\mathrm{gc(i)}}}$$

$$M^{j} = \frac{\overline{\mathrm{RD}}_{\mathrm{genome}}}{\overline{\mathrm{RD}}_{\mathrm{mappability(j)}}}$$

$$E^{k} = \frac{\overline{\mathrm{RD}}_{\mathrm{genome}}}{\overline{\mathrm{RD}}_{\mathrm{effective\ length(k)}}}$$

where $\mathrm{i, j, k}$ are the sub-intervals indices of GC-content, mappability, effective length biases; respectively. $\mathrm{RD}_{\mathrm{raw}}^{\mathrm{i,j,k}}$ is the raw RD signal of a bin, $\mathrm{RD}_{\mathrm{corrected}}^{\mathrm{i,j,k}}$ is the corrected RD signal of this bin, and $\overline{\mathrm{RD}}_{\mathrm{genome}}$ is the genome-wide mean of the RD signal. $\overline{\mathrm{RD}}_{\mathrm{gc(i)}}, \overline{\mathrm{RD}}_{\mathrm{mappability\ (j)}},$ $and\ \overline{\mathrm{RD}}_{\mathrm{effective\ length\ (k)}}$ are the average RD signals over all bins with the GC-content, mappability, and effective length as in sub-intervals i, j and k, respectively. $G^{i}, M^{j}, and\ E^{k}$ are the correction factors for GC-content, mappability, and effective length of a bin in sub-intervals i, j and k, respectively. Finally, we evaluated our interval normalization method on Hi-C datasets of different normal and cancer cell lines (Extended Fig. 1). After normalization, the RD signal become uncorrelated with the systematic biases (Extended Fig. 1).

3.3 Computing the contact frequencies

We use the valid pairs of Hi-C/3C-seq for computing the interaction frequency between all loci of the genome and constructing the contact map (genome-wide heatmap). Valid pairs are filtered to keep only read pairs within the MML from the restriction sites. Then, we bin the filtered reads at a user-defined resolution (default bin size = 100 kb). Reads are assigned to bins based on the midpoint of the restriction fragments [8]. Interaction frequency per bin is influenced by GC-content, mappability, restriction fragment length, and copy number variations. Therefore, we compute these four bias features for normalizing the contact map.

The GC-content, mappability, and effective-length scores for contact maps are computed following the HiCNorm method [9].

3.4 Correction of the contact frequencies

HiCNAtra corrects the contact map from the systematic biases and the CN-driven bias by utilizing GLM for *cis* and *trans* contact maps separately as explained in the Methods section.

Extended Figure 1

Interval normalization successfully normalized the RD signal of normal/cancer Hi-C data from the systematic biases. Bar charts showing the mean read counts before (blue) and after (red) normalization for each systematic bias (effective length, mappability, and GC-content). The interval of bias values is divided into 20 equally spaced sub-intervals and the average RD signal over all bins within each sub-interval are plotted. The bar charts are plotted for GM12878 (a), IMR90 (b), MCF7 (c), and LNCaP (d) Hi-C datasets.

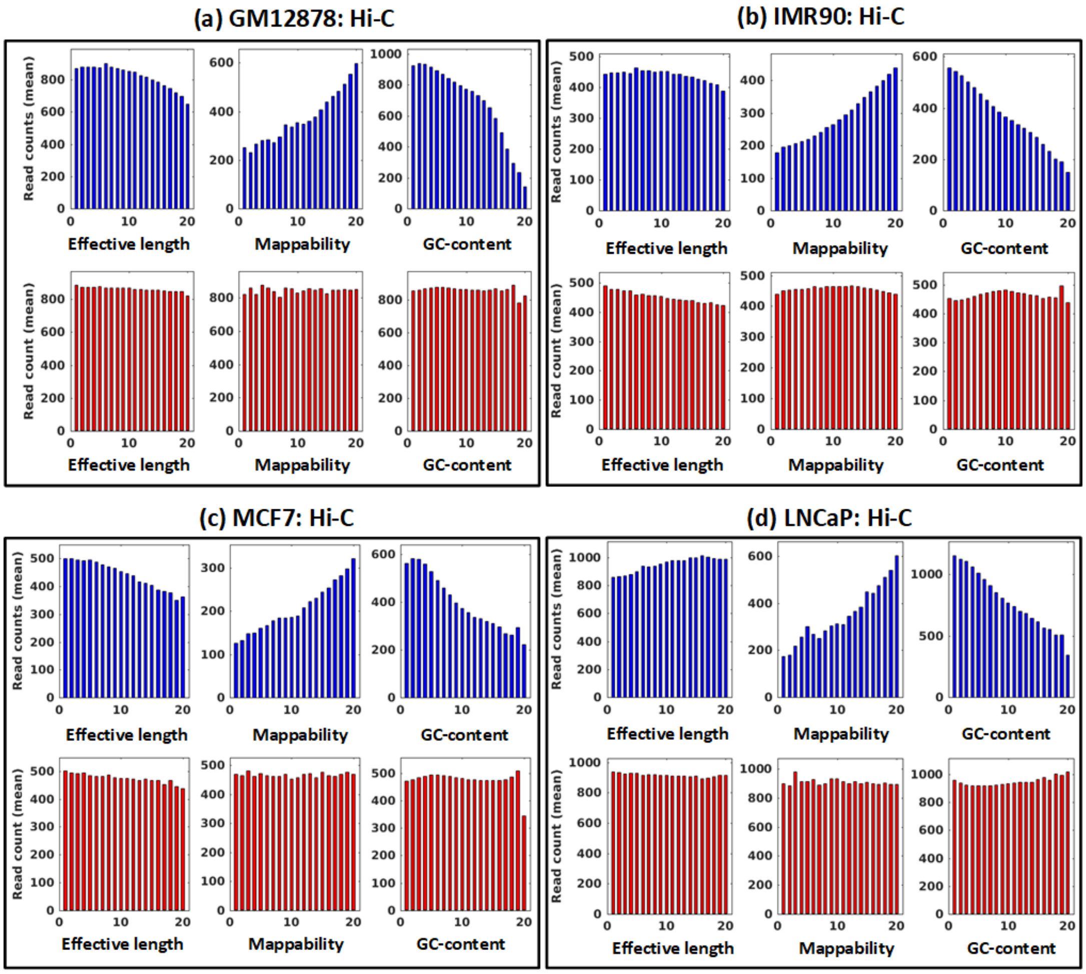

References

1. Naumova, N., et al., *Analysis of long-range chromatin interactions using Chromosome Conformation Capture.* Methods, 2012. **58**(3): p. 192-203.
2. Ferraiuolo, M.A., et al., *From cells to chromatin: capturing snapshots of genome organization with 5C technology.* Methods, 2012. **58**(3): p. 255-67.
3. van Berkum, N.L. and J. Dekker, *Determining spatial chromatin organization of large genomic regions using 5C technology.* Methods Mol Biol, 2009. **567**: p. 189-213.
4. Karimzadeh, M., et al., *Umap and Bismap: quantifying genome and methylome mappability.* Nucleic Acids Res, 2018.
5. Miller, C.A., et al., *ReadDepth: a parallel R package for detecting copy number alterations from short sequencing reads.* PLoS One, 2011. **6**(1): p. e16327.
6. Khalil, A.I.S., et al., *Hierarchical discovery of large-scale and focal copy number alterations in low-coverage cancer genomes.* BMC Bioinformatics, 2020. **21**(1): p. 147.
7. Abyzov, A., et al., *CNVnator: an approach to discover, genotype, and characterize typical and atypical CNVs from family and population genome sequencing.* Genome Res, 2011. **21**(6): p. 974-84.
8. Imakaev, M., et al., *Iterative correction of Hi-C data reveals hallmarks of chromosome organization.* Nat Methods, 2012. **9**(10): p. 999-1003.
9. Hu, M., et al., *HiCNorm: removing biases in Hi-C data via Poisson regression.* Bioinformatics, 2012. **28**(23): p. 3131-3.

SUPPLEMENTARY FIGURES

Figure S1

Comparison of genome-wide copy number profile (normalized RD signal) computed from Hi-C data using exact-cut, midpoint and entire-fragment counting approaches. The copy number profiles of karyotypically-normal lymphoblastoid (GM12878) (a) and LNCaP (b) cell lines are plotted. Each grey dot represents the copy number of a bin. The red arrows indicate the variation levels of chr 3, which is free of LCVs. Both GM12878 and LNCaP cells showed that signal variation levels of entire-fragment approach are significantly less than exact-cut and midpoint approaches.

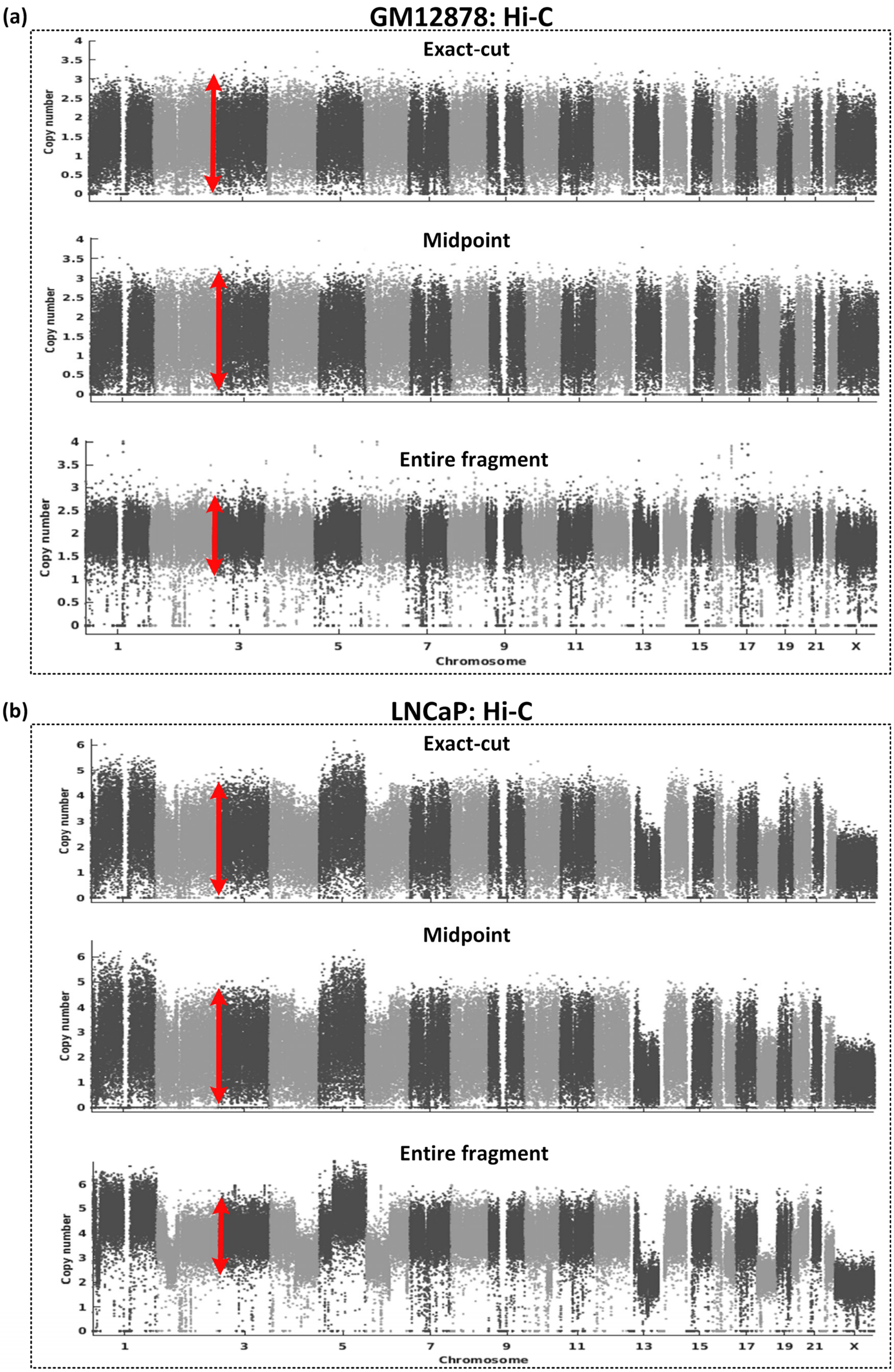
(a)
GM12878: Hi-C
Exact-cut
Midpoint
Entire fragment
Copy number
Chromosome
1
3
5
7
9
11
13
15
17
19
21
X
(b)
LNCaP: Hi-C
Exact-cut
Midpoint
Entire fragment
Copy number
Chromosome

Figure S2

Visual comparison of the copy number profiles of hyperploid MCF7 breast cancer cell line identified by HiCNAtra using midpoint, exact-cut, and entire fragment approaches. Coverage plots showing CNV profiles of chr1: 80-100Mb estimated by entire-fragment (first panel), midpoint (second panel) and exact-cut (third panel) from MCF7 Hi-C data. The last panel shows the CNAtra-estimated CNV profiles derived from WGS data of MCF7. CNAtra performs additional filtering step to exclude homozygous deletions (such as CNV1) in regions with low mappability or that belong to blacklisted or gap regions. Each grey dot represents the copy number of a bin. The red line represents the copy number track where any amplitude transition indicates a new CNV region. The red blocks indicate false negative CNVs that are missed or wrongly called by midpoint or exact-cut approaches compared to entire-fragment counting approach respectively.

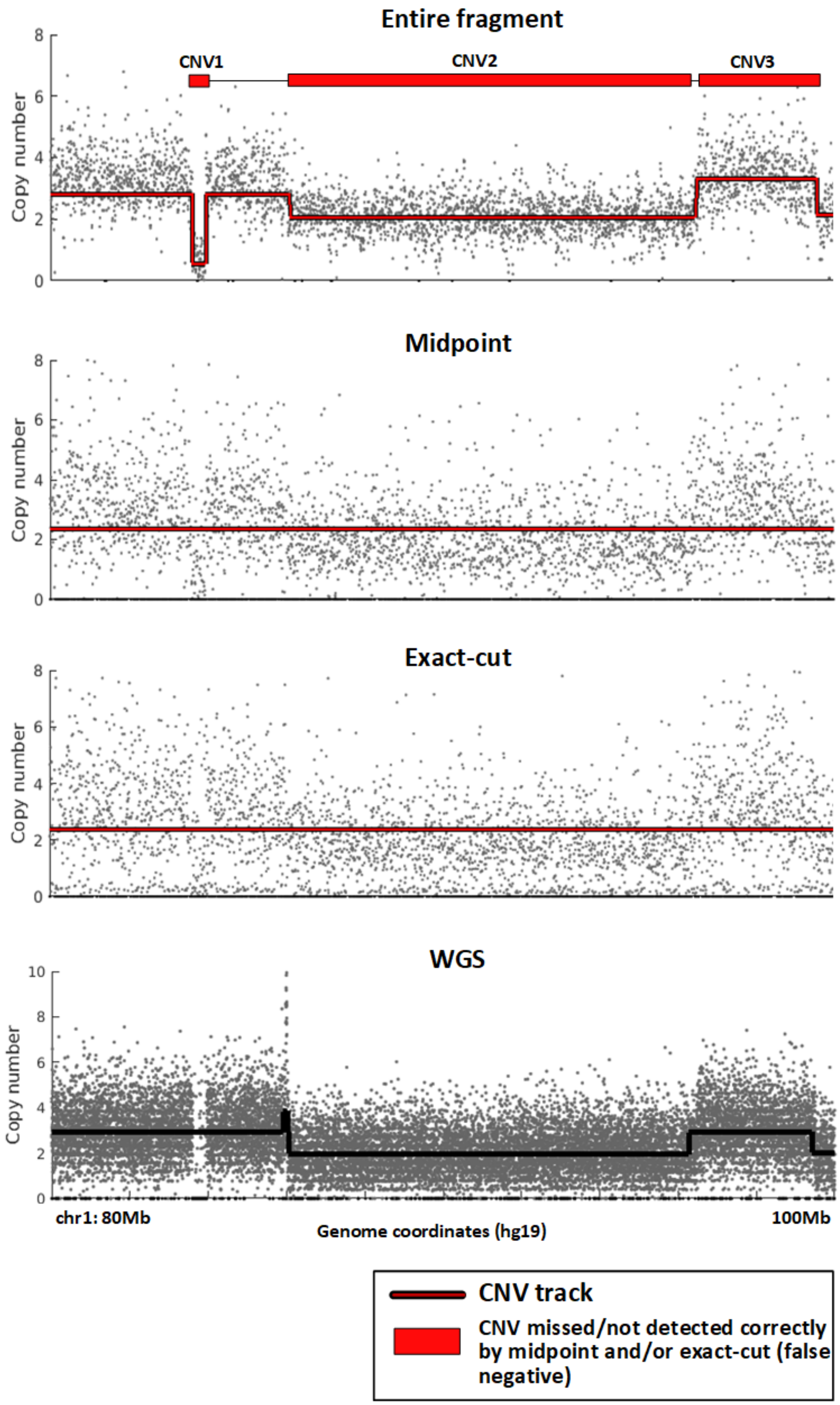
Entire fragment
CNV1
CNV2
CNV3
Copy number
Midpoint
Copy number
Exact-cut
Copy number
WGS
Copy number
chr1: 80Mb
Genome coordinates (hg19)
100Mb
CNV track
CNV missed/not detected correctly by midpoint and/or exact-cut (false negative)

Figure S3

Visual comparison of the copy number profile of hypotetraploid LNCaP cancer cell line identified by HiCNAtra using midpoint, exact-cut, and entire-fragment approaches. Coverage plots showing CNV profiles of chr10: 85-120Mb estimated by entire-fragment (first panel), midpoint (second panel) and exact-cut (third panel) from LNCaP Hi-C data. The last panel shows the CNAtra-estimated CNV profiles derived from WGS data of LNCaP. CNAtra performs additional filtering step to exclude homozygous deletions in regions with low mappability or that belong to blacklisted or gap regions. Each grey dot represents the copy number of a bin. The red line represents the copy number track where any amplitude transition indicates a new CNV region. The blue blocks show true positive CNVs from the RD signal computed by the three approaches. The red block indicates false negative CNV that are missed or wrongly called by midpoint or exact-cut approaches compared to entire-fragment counting approach respectively.

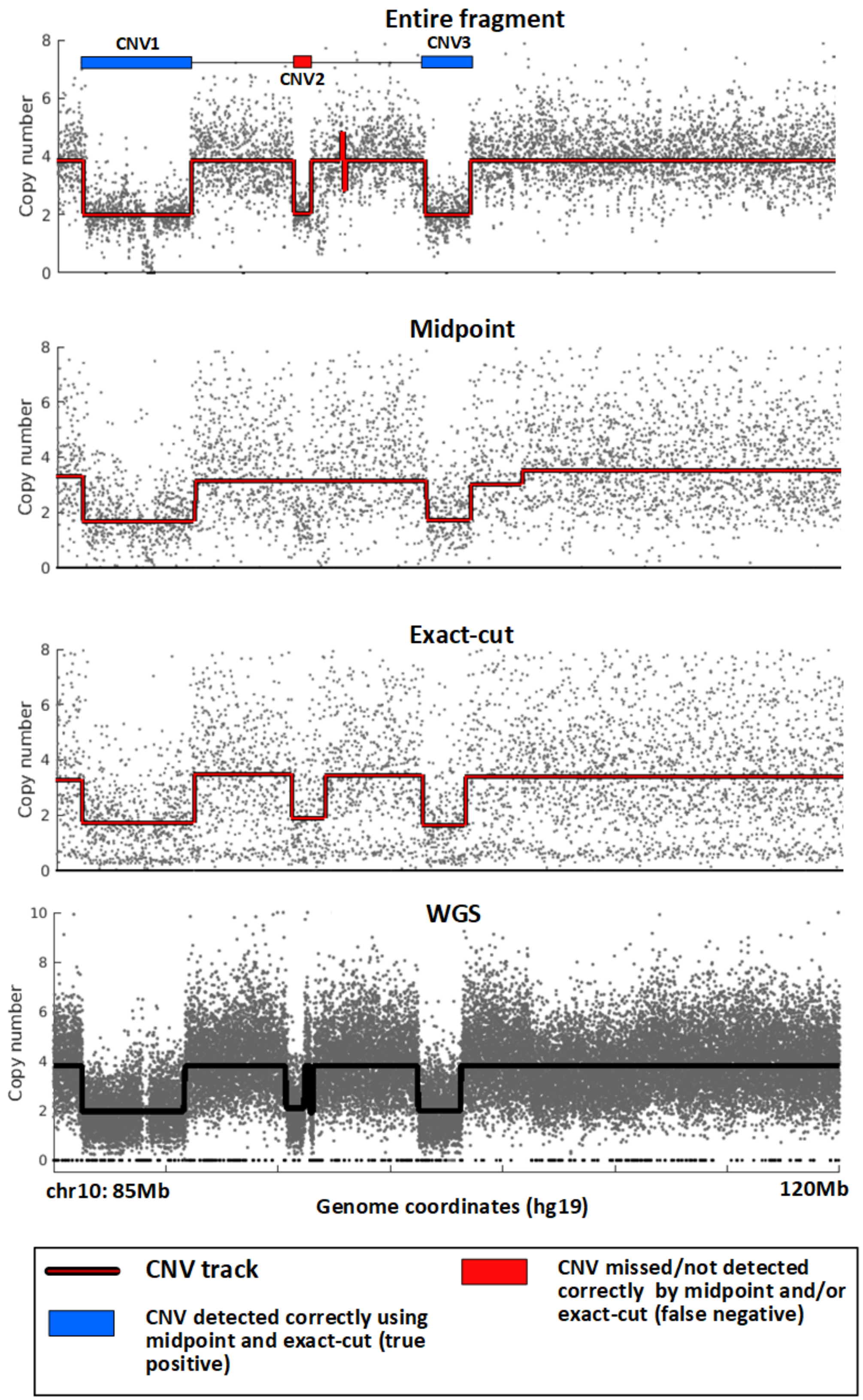

Entire fragment
CNV1
CNV2
CNV3
Copy number
Midpoint
Copy number
Exact-cut
Copy number
WGS
Copy number
chr10: 85Mb
120Mb
Genome coordinates (hg19)
CNV track
CNV detected correctly using midpoint and exact-cut (true positive)
CNV missed/not detected correctly by midpoint and/or exact-cut (false negative)

Figure S4

Visual comparison of genome-wide RD signal computed from Hi-C data and WGS data from same cell line. The genome-wide coverage plot (chr 1- chr X) of MCF7 (a) and LNCaP (b) cancer cell lines computed by midpoint (top panel), exact-cut (second panel) and entire fragment approach (third panel) from Hi-C data as well as WGS data (bottom panel). WGS reads are obtained from input control of ChIP-seq experiment of the corresponding cell line.

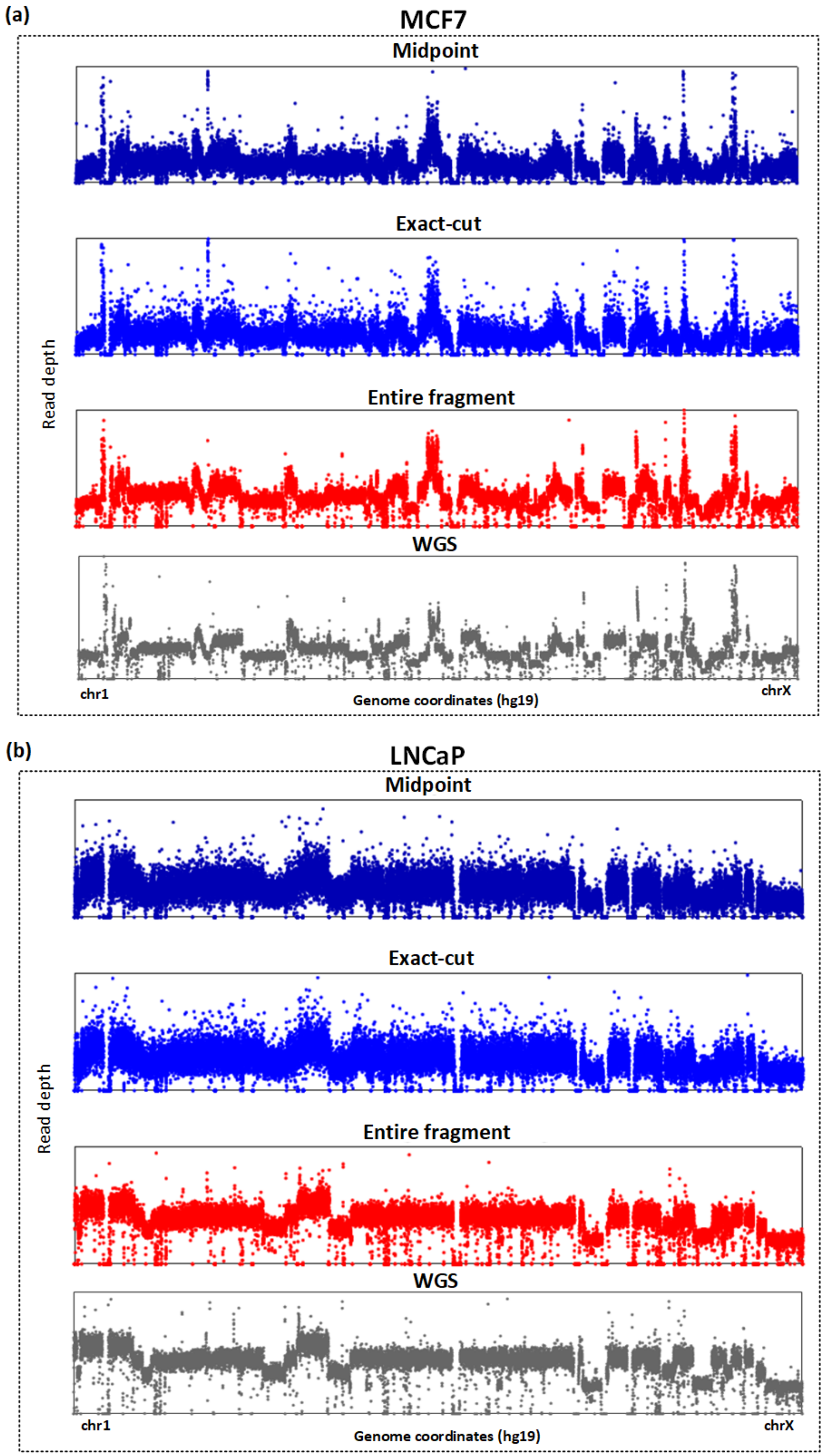
(a)
MCF7
Midpoint
Exact-cut
Entire fragment
WGS
Read depth
chr1
Genome coordinates (hg19)
chrX
(b)
LNCaP
Midpoint
Exact-cut
Entire fragment
WGS
Read depth
chr1
Genome coordinates (hg19)
chrX

Figure S5

Visual comparison of genome-wide RD signal computed from Hi-C/3C-seq data versus WGS data. The genome-wide coverage plot (chr 1 - chr X) of LNCaP (a) and K562 (b) cancer cells computed from Hi-C/3C-seq data (top panel) and WGS data (bottom panel). WGS reads are obtained from input control of ChIP-seq experiment of the corresponding cell line. Each red dot is the RD value per bin computed from Hi-C/3C-seq data whereas each grey dot represents RD value per bin computed from WGS data.

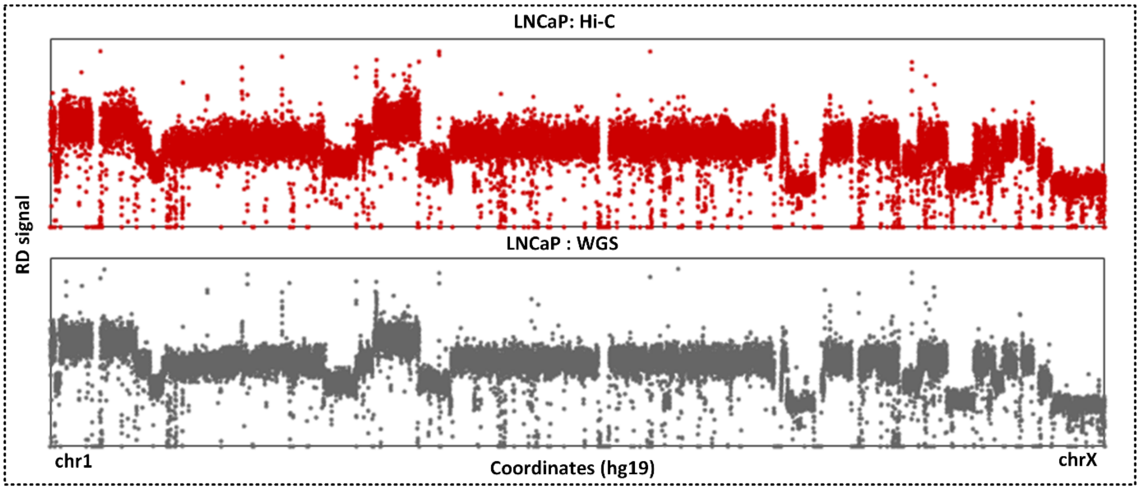
LNCaP: Hi-C
LNCaP : WGS
RD signal
chr1
Coordinates (hg19)
chrX
(a)

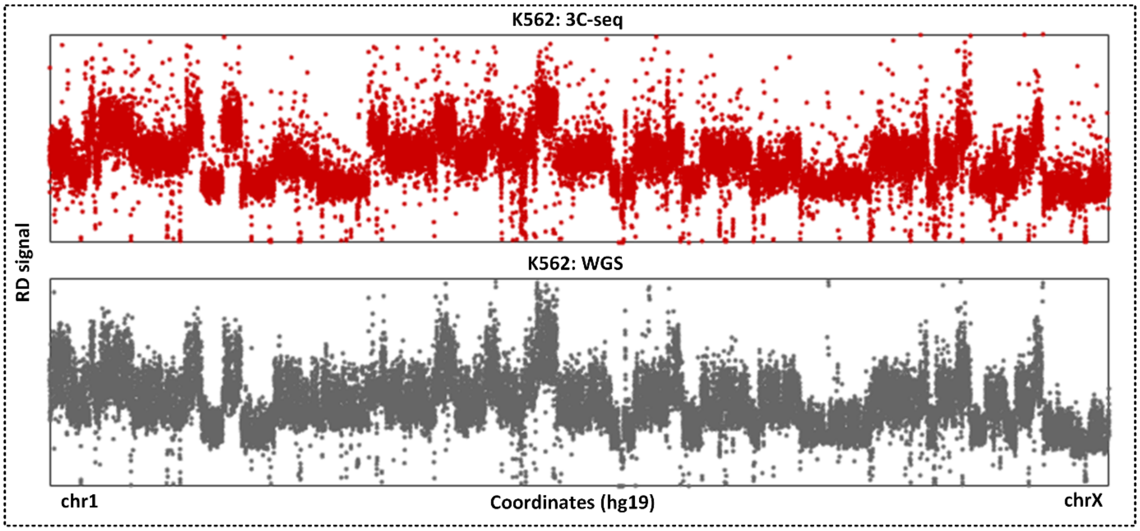
K562: 3C-seq
K562: WGS
RD signal
chr1
Coordinates (hg19)
chrX
(b)

Figure S6

RD frequency distributions from Hi-C/3C-seq data of normal and cancer cell lines. (a) Cancer cell lines (MCF7, LNCaP, PC3, H69AR and K562) exhibit a multimodal RD frequency distribution. (b) Normal cell lines (GM12878, IMR90 and PrEC) show a unimodal RD frequency distribution. The peak at CN = 1 in male-derived PrEC cells is due to the single X chromosome.

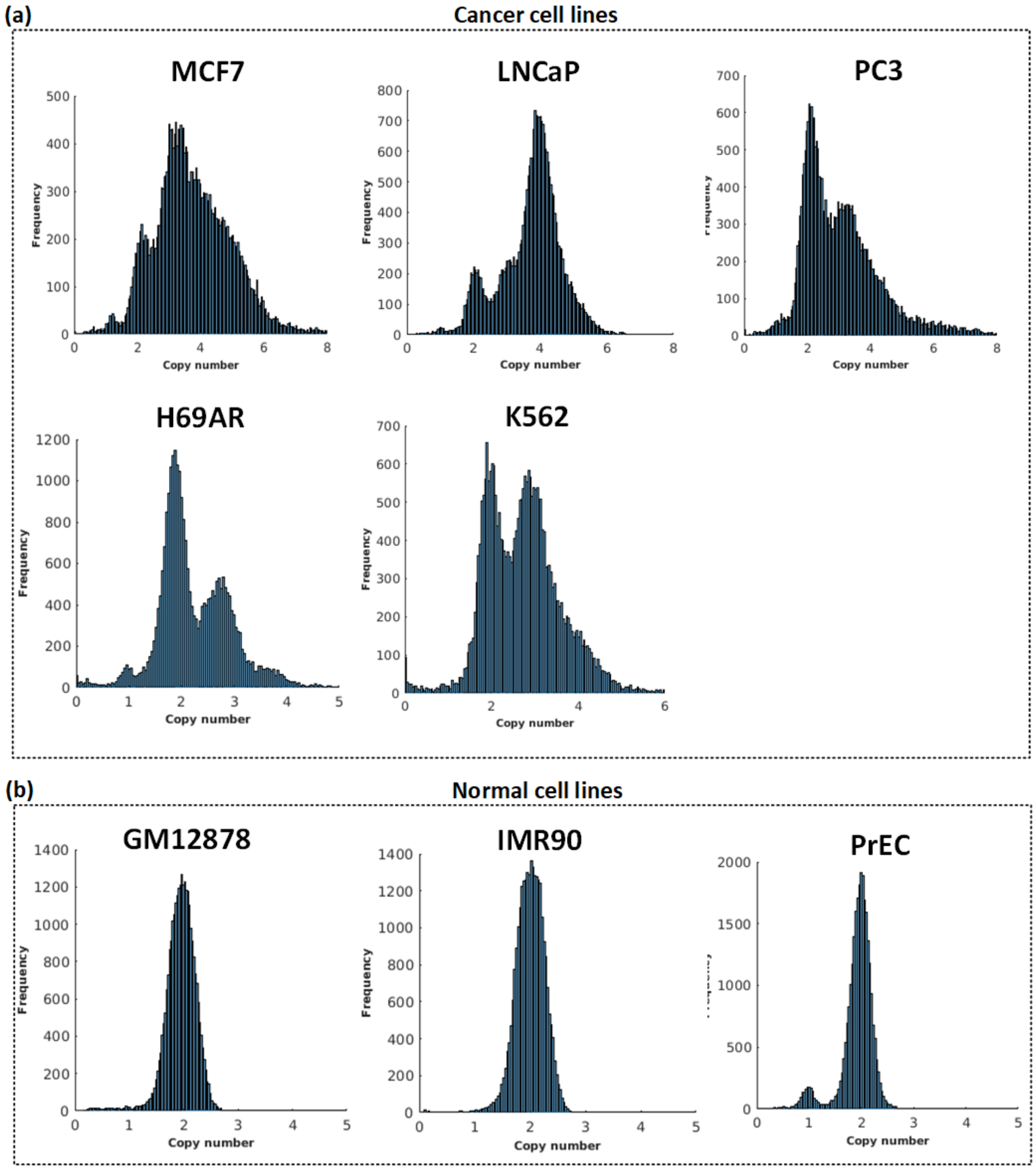
(a)
Cancer cell lines
MCF7
LNCaP
PC3
H69AR
K562
Frequency
Copy number
(b)
Normal cell lines
GM12878
IMR90
PrEC
Frequency
Copy number

Figure S7

Numbers and widths (sizes) of large-scale copy number variations (LCVs) and focal alterations (FAs) estimated by HiCNAtra in different cell lines. Box plot of the width (in Mb) of LCVs (a) and FAs (b) estimated from Hi-C/3C-seq data are shown. The numbers of LCVs and FAs are denoted at the bottom of the box plot for each cell line. The LCVs detected in the normal cell lines (GM12878 and IMR90) are located at the vicinity (boundary) of the telomeres.

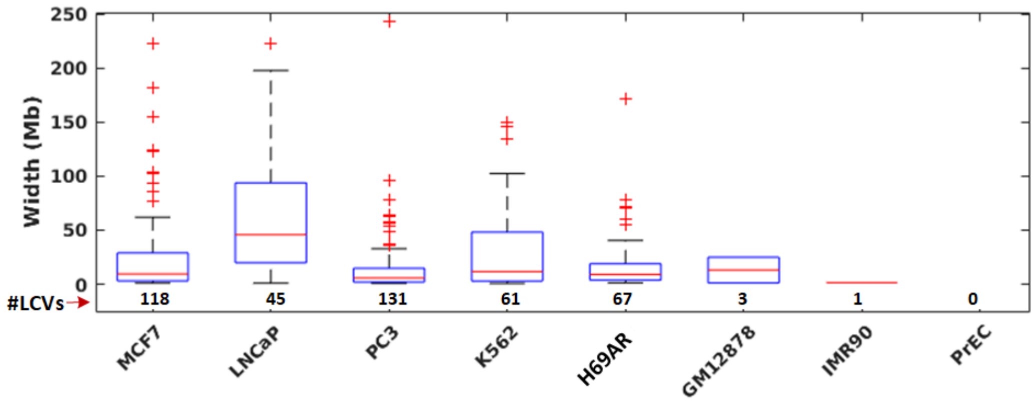

(a) Large-scale copy number variations

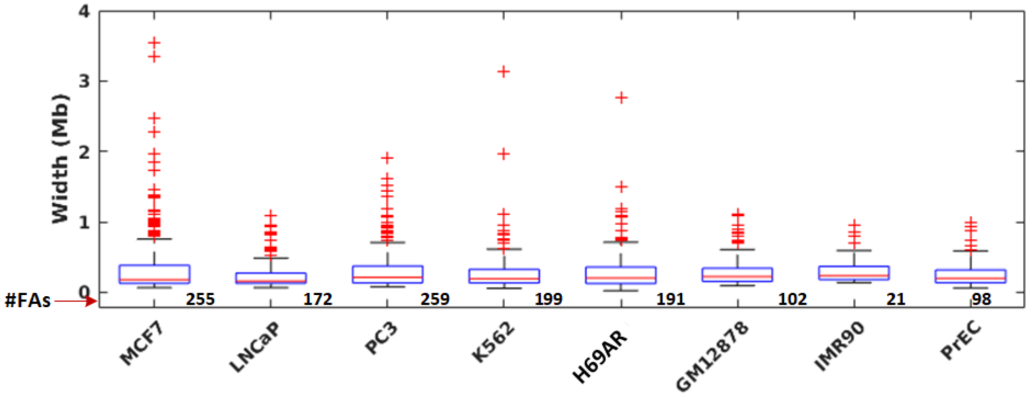

(b) Focal alterations

Figure S8

Visual comparison of the copy number profiles identified by CNV detection module of OneD, HiNT and HiCNAtra. CNV tracks of (a) MCF7 and (b) PC3 cancer cell lines are plotted from Hi-C data estimated by OneD (top panel), HiNT (middle panel) and HiCNAtra (bottom panel). Each grey dot represents the copy number of a bin. The black line represents the copy number track where any amplitude transition indicates a new CNV region. For HiCNAtra, the copy number track is computed from the LCVs only.

(a)

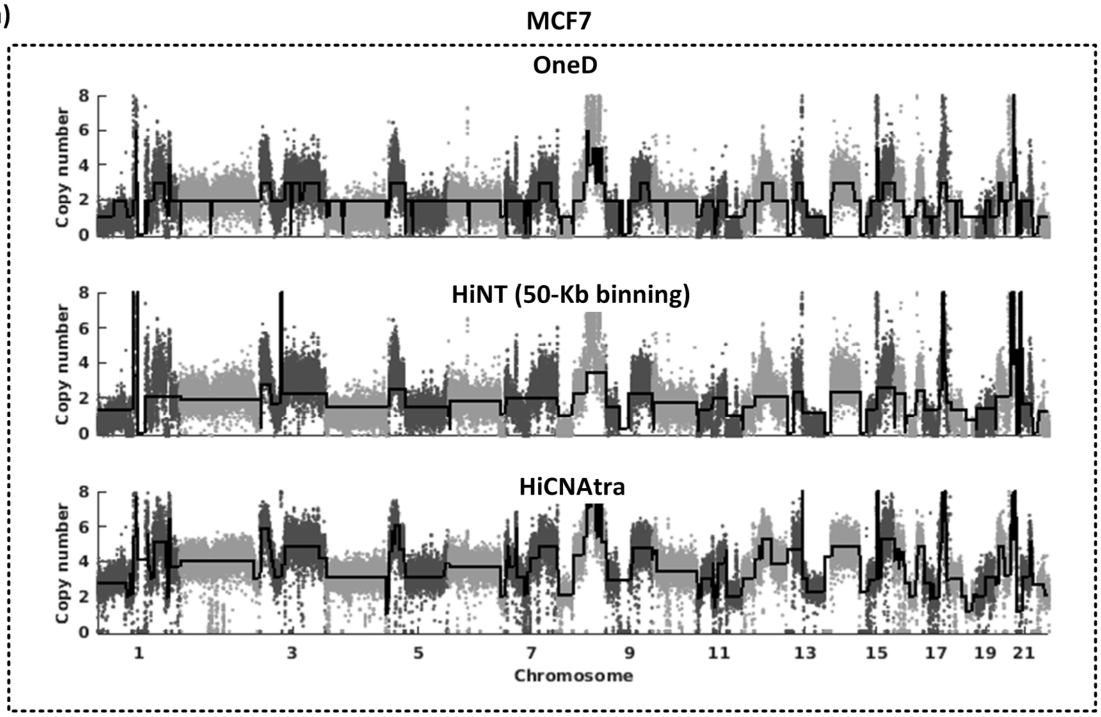

MCF7
OneD
HiNT (50-Kb binning)
HiCNAtra
Copy number
Chromosome

(b)

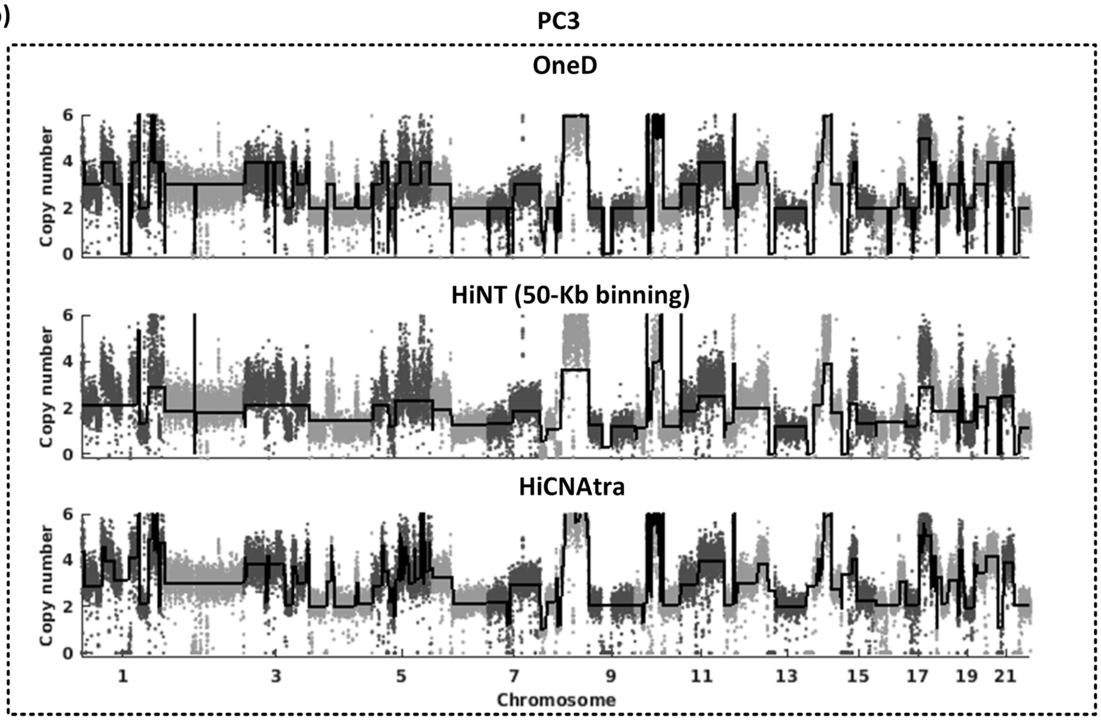

PC3
OneD
HiNT (50-Kb binning)
HiCNAtra
Copy number
Chromosome

Figure S9

Visual comparison of the copy number profiles of MCF7 cancer cell line identified by CNV detection module of OneD, HiNT and HiCNAtra. The CNV tracks of chr 4 (a) and chr 18 (b) generated by OneD (top panel), HiNT with 5-kb binning (second panel), HiNT with 50-kb binning (third panel), and HiCNAtra (bottom panel) from MCF7 Hi-C data. Each grey dot represents the copy number of a bin. The black line represents the copy number track where any amplitude transition indicates a new CNV region. The green vertical lines denote the boundaries of centromere and telomeres. In the bottom panel (HiCNAtra output), the red lines represent the focal alterations and the blue bins indicate focally amplified/deleted regions. On top of each panel, the blue, red and black blocks indicate true positive, false negative and false negative CNVs, respectively. It is visually clear that both OneD and HiNT wrongly estimate the copy number of most regions as 2 for MCF7 cells with modal chromosome number = 82.

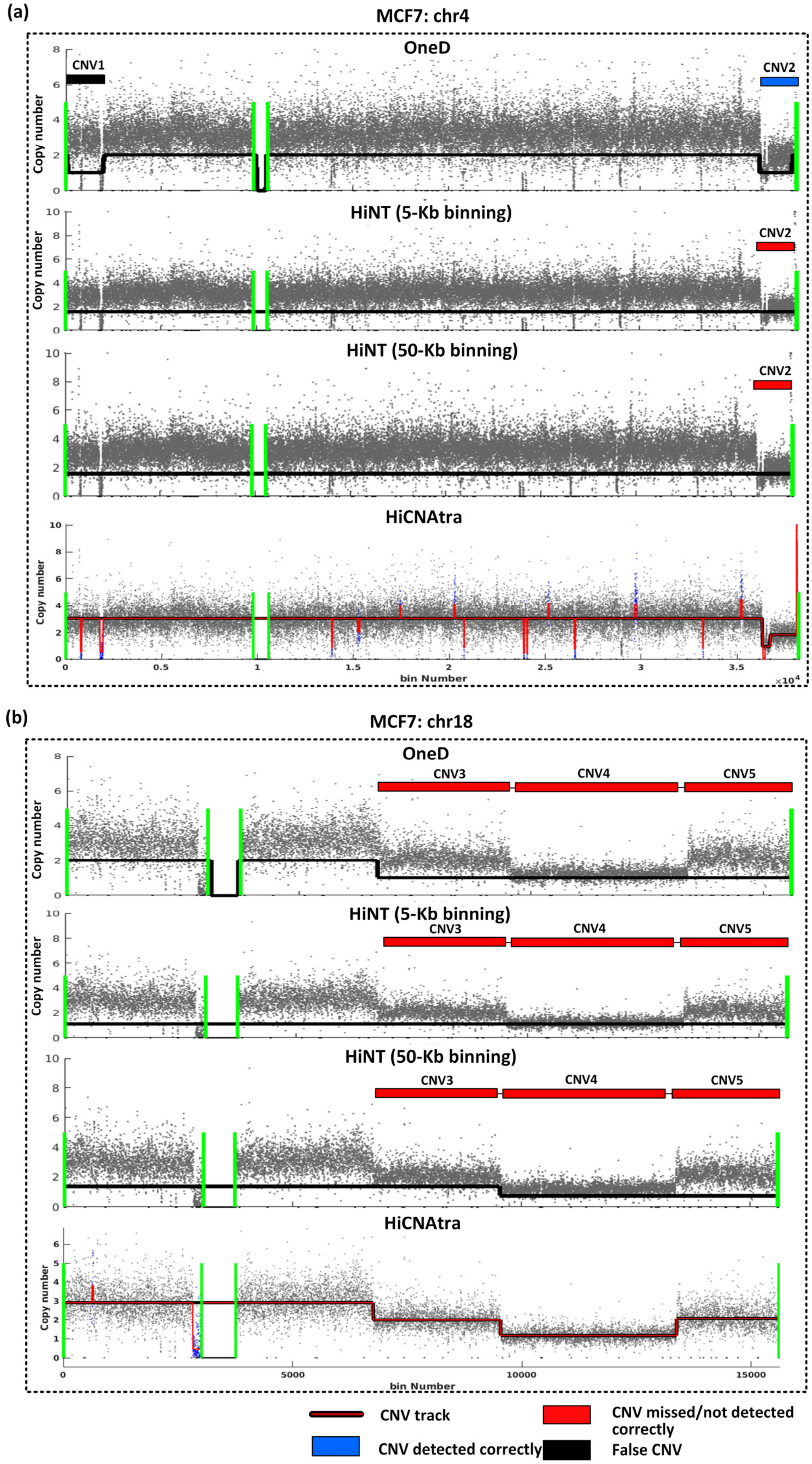
(a)
MCF7: chr4
OneD
CNV1
CNV2
Copy number
HiNT (5-Kb binning)
CNV2
Copy number
HiNT (50-Kb binning)
CNV2
HiCNAtra
Copy number
bin Number
×10^4
(b)
MCF7: chr18
OneD
CNV3
CNV4
CNV5
Copy number
HiNT (5-Kb binning)
CNV3
CNV4
CNV5
Copy number
HiNT (50-Kb binning)
CNV3
CNV4
CNV5
HiCNAtra
Copy number
bin Number
CNV track
CNV missed/not detected correctly
CNV detected correctly
False CNV

Figure S10

Visual comparison of the copy number profile from 3C-seq data of H69AR (a) and K562 (b) cancer cell lines identified by OneD, HiNT and HiCNAtra. Each grey dot represents the copy number of a bin. The black line represents the copy number track where any amplitude transition indicates a new CNV region. For HiCNAtra, the copy number track is computed from the LCVs only.

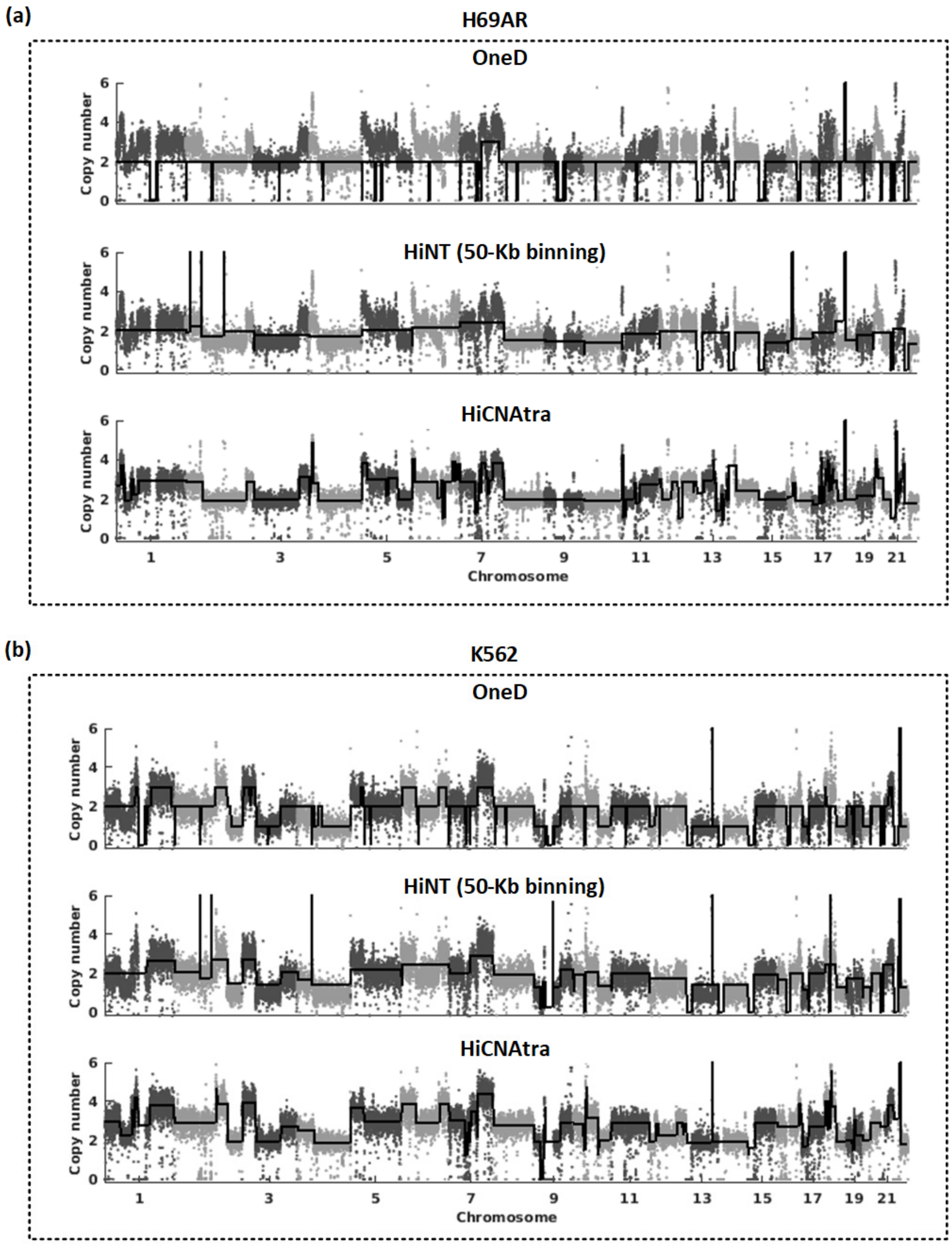
(a)
H69AR
OneD
HiNT (50-Kb binning)
HiCNAtra
Copy number
0
2
4
6
1
3
5
7
9
11
13
15
17
19
21
Chromosome
(b)
K562
OneD
HiNT (50-Kb binning)
HiCNAtra
Copy number
0
2
4
6
1
3
5
7
9
11
13
15
17
19
21
Chromosome

Figure S11

Visual comparison of the copy number profile of H69AR cancer cell line identified by CNV detection module of OneD, HiNT and HiCNAtra. The CNV track of chr 13 (a) and chr 16 (b) generated by OneD (top panel), HiNT with 5-kb binning (second panel), HiNT with 50-kb binning (third panel) and HiCNAtra (bottom panel) from H69AR 3C-seq data. Each grey dot represents the copy number of a bin. The black line represents the copy number track where any amplitude transition indicates a new CNV region. The green vertical lines denote the boundaries of centromere and telomeres. In the bottom panel (HiCNAtra output), the red lines represent the focal alterations and the blue bins indicate focally amplified/deleted regions. On top of each panel, the blue, red and black blocks indicate CNVs correctly, missed and falsely detected CNVs, respectively based on visual inspection of RD signal.

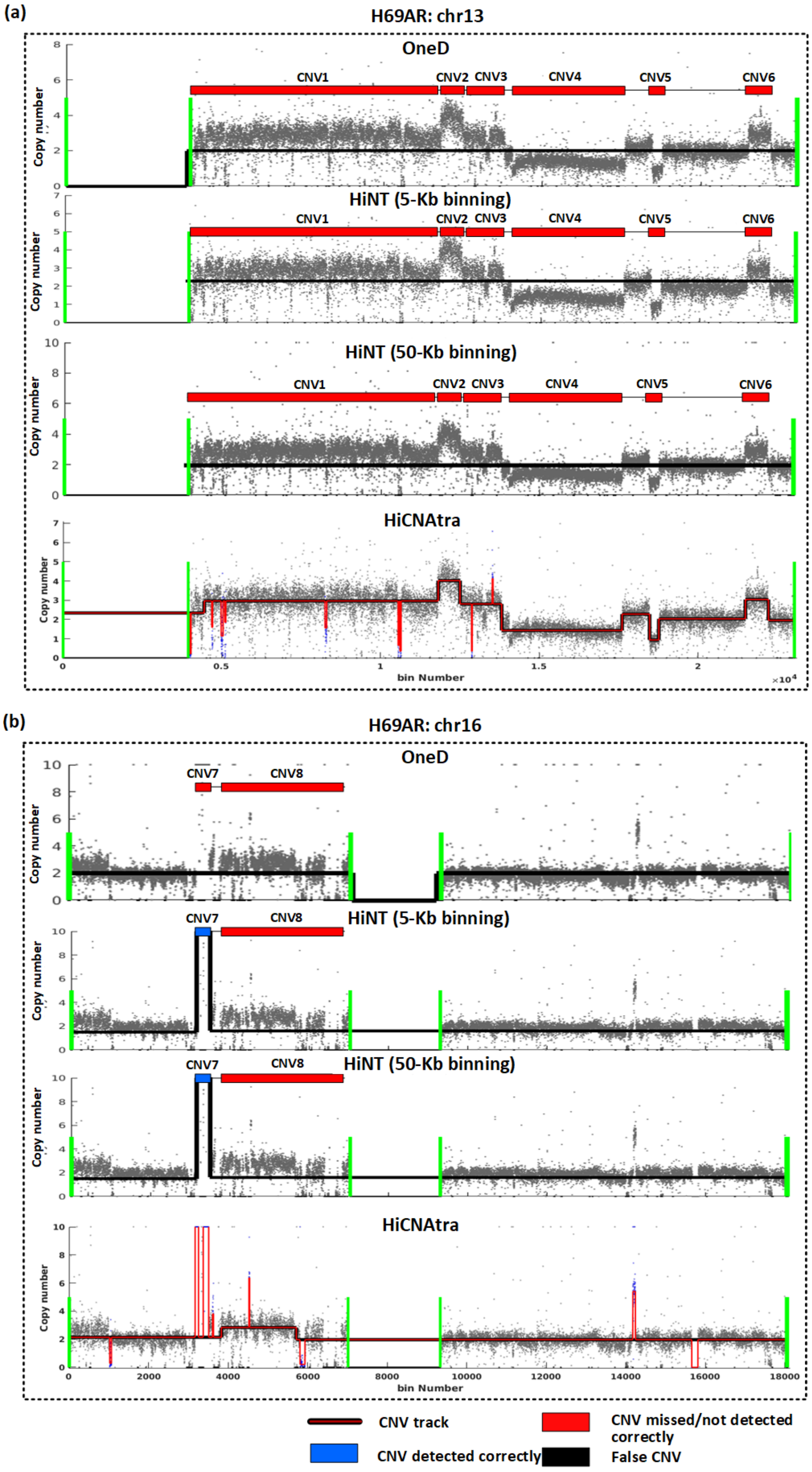
(a)
H69AR: chr13
OneD
HiNT (5-Kb binning)
HiNT (50-Kb binning)
HiCNAtra
CNV1
CNV2
CNV3
CNV4
CNV5
CNV6
Copy number
bin Number
(b)
H69AR: chr16
OneD
HiNT (5-Kb binning)
HiNT (50-Kb binning)
HiCNAtra
CNV7
CNV8
Copy number
bin Number
CNV track
CNV detected correctly
CNV missed/not detected correctly
False CNV

Figure S12

Visual illustration of the RD signal and CNV profile estimated by HiNT and CNV caller module of HiCNAtra. Genome-wide RD signal and CNV profiles generated by HiNT (top panel) and HiCNAtra (bottom panel) from Hi-C data of MCF7 (a) and LNCaP (b), and from 3C-seq data of H69AR (c) are plotted. In HiNT output (top panel), the black dots represent the copy number of each bin whereas the red line is the copy number of each segment. In HiCNAtra output (bottom panel), each grey dot represents the copy number of a bin and the black line represents the copy number track where any amplitude transition indicates a new CNV region. Plots show that HiNT can correctly estimate the RD signal of Hi-C data of MCF7 and LNCaP, however it still identifies some false negative CNVs due to the multimodality feature of RD signal in cancer genomes. HiNT's inaccurate estimate of the RD signal of H69AR 3C-seq data results in false negative CNVs.

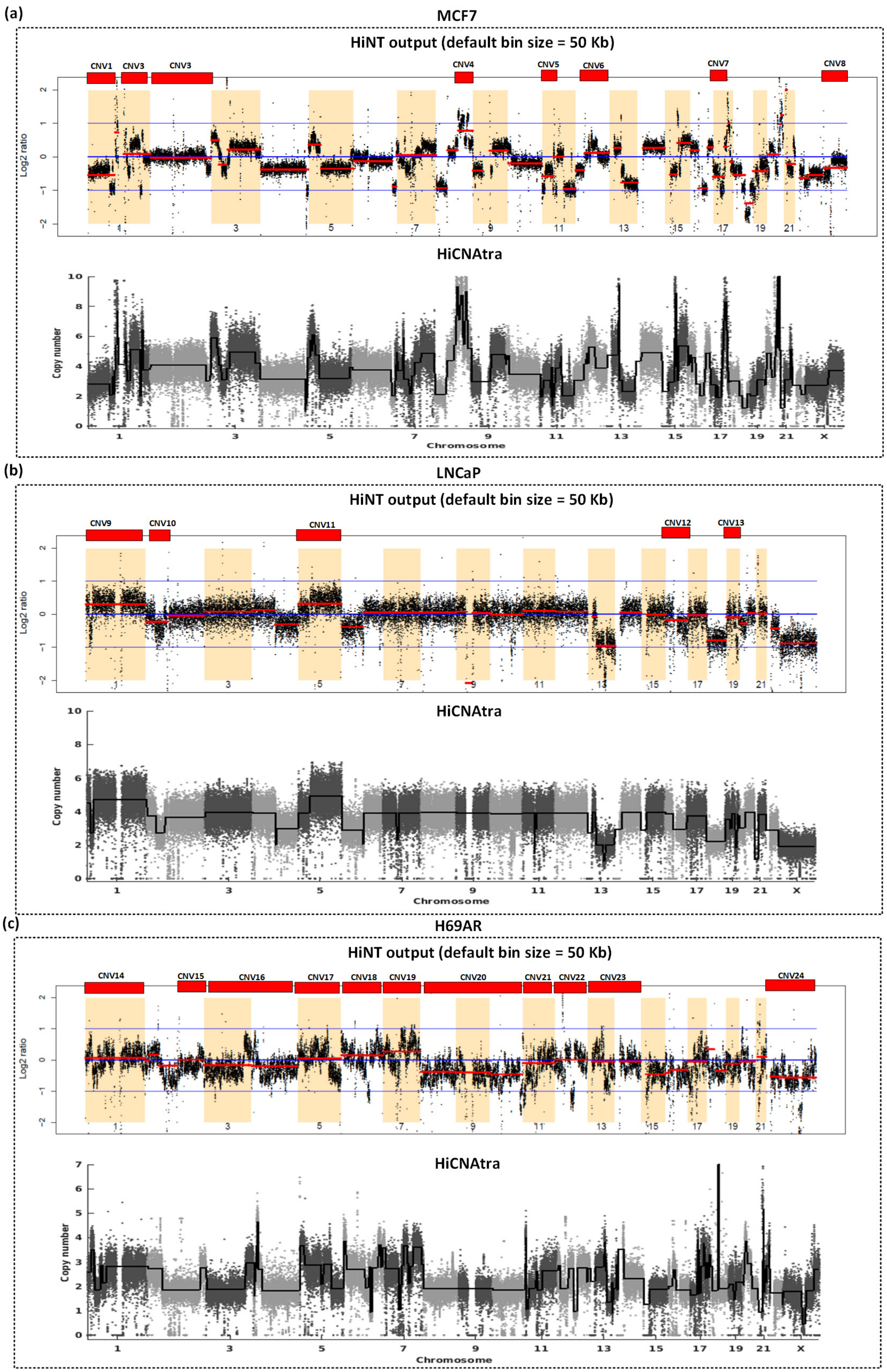

(a)
MCF7
HiNT output (default bin size = 50 Kb)
CNV1
CNV3
CNV3
CNV4
CNV5
CNV6
CNV7
CNV8
Log2 ratio
HiCNAtra
Copy number
Chromosome
(b)
LNCaP
HiNT output (default bin size = 50 Kb)
CNV9
CNV10
CNV11
CNV12
CNV13
Log2 ratio
HiCNAtra
Copy number
Chromosome
(c)
H69AR
HiNT output (default bin size = 50 Kb)
CNV14
CNV15
CNV16
CNV17
CNV18
CNV19
CNV20
CNV21
CNV22
CNV23
CNV24
Log2 ratio
HiCNAtra
Copy number
Chromosome

Figure S13

Visual illustration of the performance of Hi-C correction tools (ICE, CAIC, OneD, HiCNAtra) using MCF7 cancer cell line having aberrant karyotype. For each chromosome (chr 3, chr 9, chr 13, and chr 15), the top panel shows the CNV track computed by HiCNAtra. Each grey dot represents the copy number of a bin. The black line represents the copy number track where any amplitude transition indicates a new CNV region. The second panel shows the uncorrected (raw) contact map. The next four panels show the post-corrected contact map using ICE, CAIC, OneD and HiCNAtra tools.

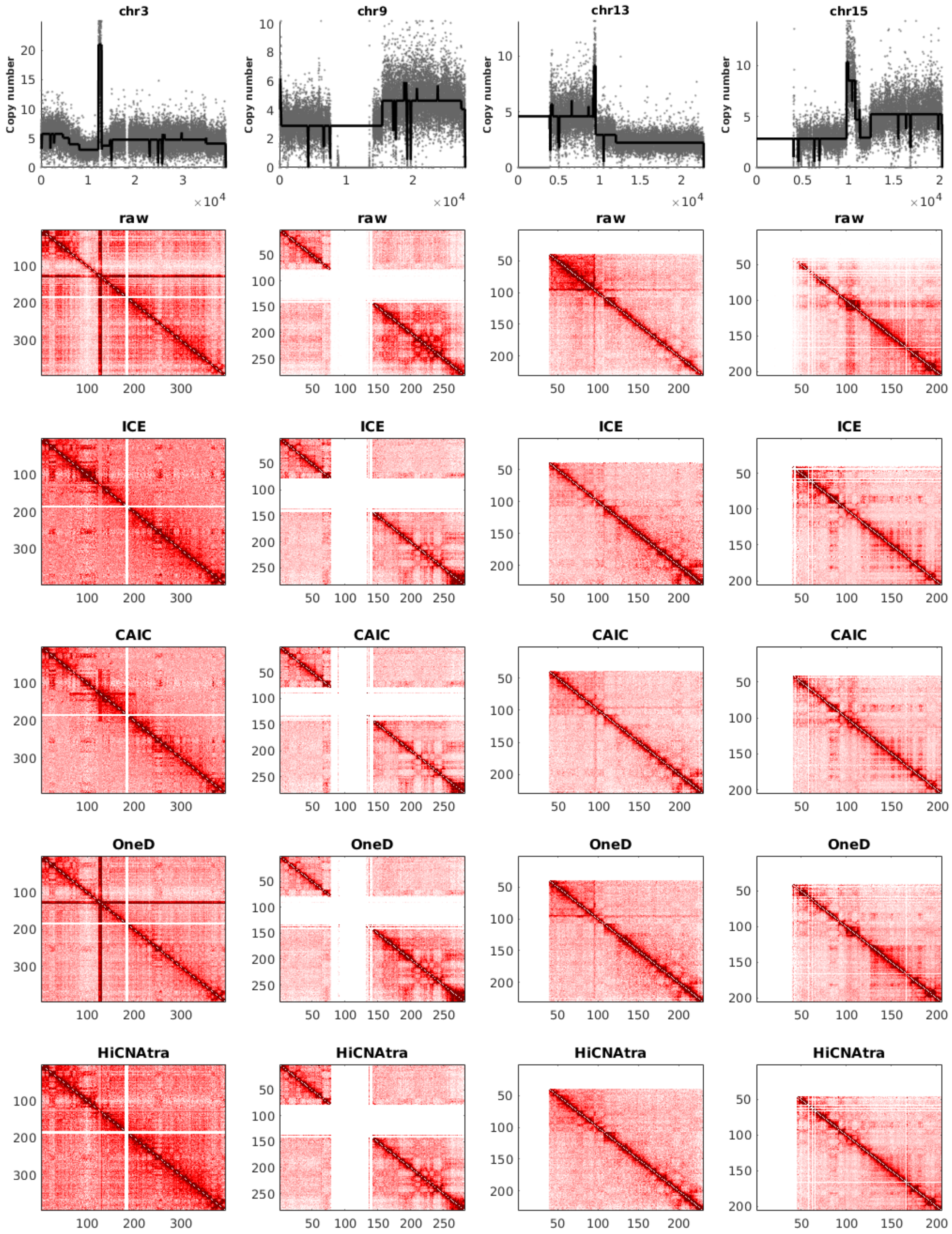

chr3
chr9
chr13
chr15
Copy number
raw
ICE
CAIC
OneD
HiCNAtra

Figure S14

Visual comparison of the Hi-C correction tools (ICE, CAIC, OneD, HiCNAtra) in attenuating the 1D signal variation of chr 9 of MCF7. The top panel shows the CNV track computed by HiCNAtra. Each grey dot represents the copy number of a bin. The black line represents the copy number track where any amplitude transition indicates a new CNV region. The second panel shows the raw (uncorrected) 1D signal. The next four panels show the post-correction 1D signal using ICE, CAIC, OneD and HiCNAtra tools. For each post-normalized 1D signal, the Spearman correlation (ρ) and standard deviation (s) are indicated next to each panel. The blue line shows the moving average of the 1D signal.

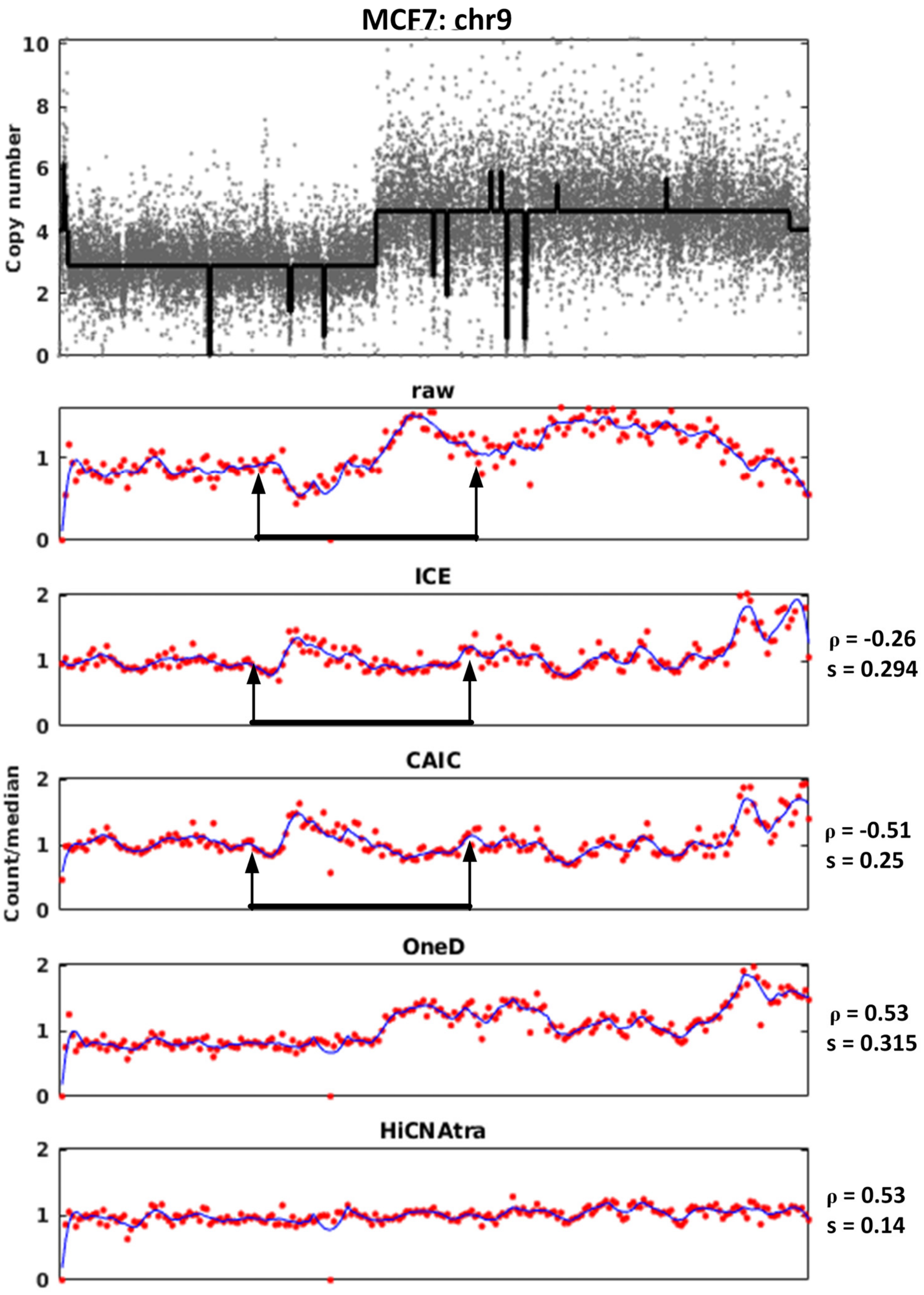

MCF7: chr9
Copy number
0
2
4
6
8
10
raw
0
1
ICE
0
1
2
ρ = -0.26
s = 0.294
CAIC
Count/median
0
1
2
ρ = -0.51
s = 0.25
OneD
0
1
2
ρ = 0.53
s = 0.315
HiCNAtra
0
1
2
ρ = 0.53
s = 0.14

Figure S15

Visual comparison of the Hi-C correction tools (ICE, CAIC, OneD, HiCNAtra) in attenuating the 1D signal variation of chr 2-8 and chr 10 of MCF7. For each chromosome, the top panel shows the CNV track computed by HiCNAtra. Each grey dot represents the copy number of a bin. The red line represents the copy number track where any amplitude transition indicates a new CNV region. The second panel shows the raw (uncorrected) 1D signal. The next four panels show the post-correction 1D signal using ICE, CAIC, OneD and HiCNAtra tools. The blue line shows the moving average of the 1D signal.

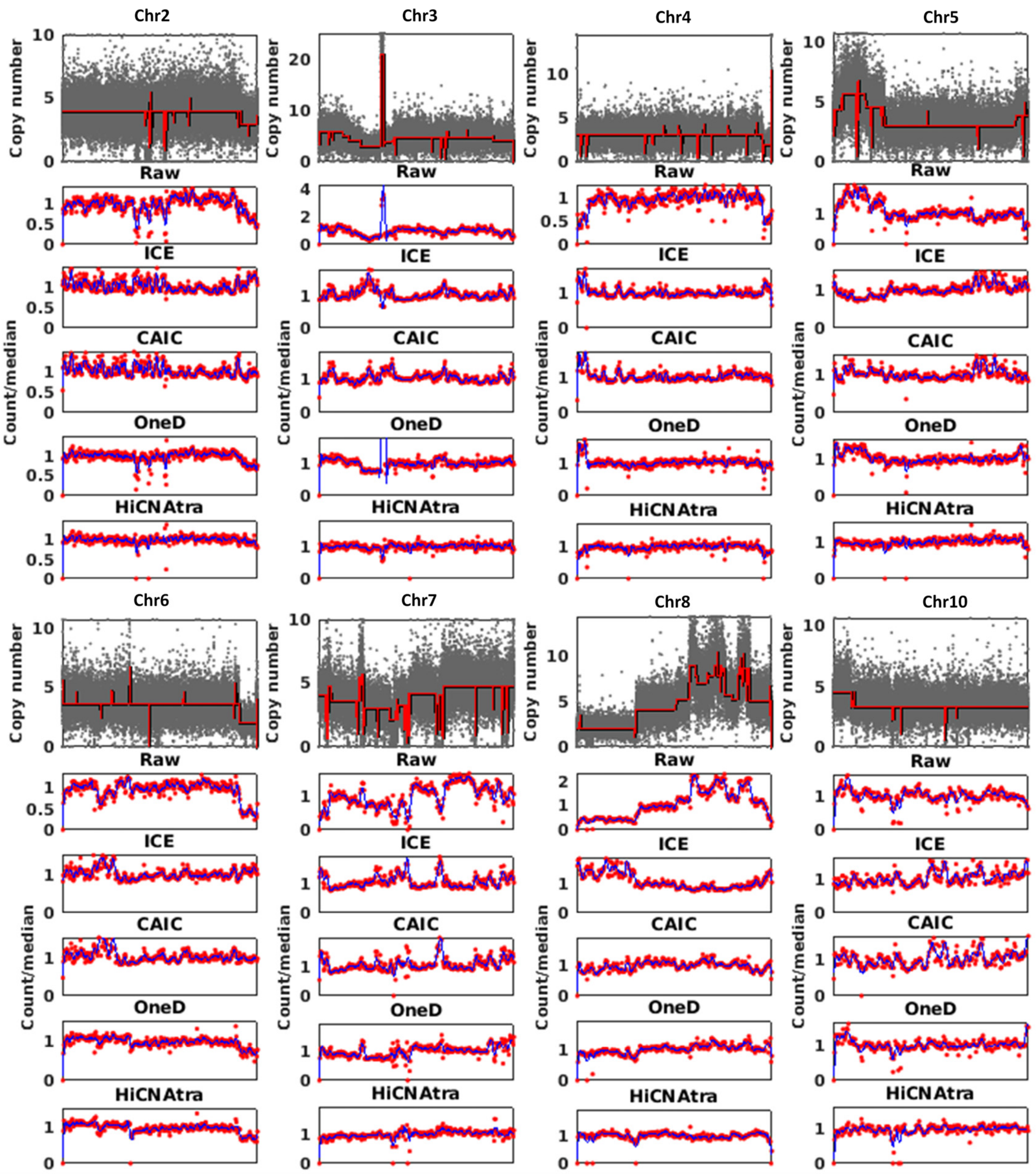

Chr2
Chr3
Chr4
Chr5
Copy number
Raw
ICE
CAIC
OneD
HiCNAtra
Count/median
Chr6
Chr7
Chr8
Chr10
Copy number
Raw
ICE
CAIC
OneD
HiCNAtra
Count/median

Figure S16

Visual comparison of the Hi-C correction tools (ICE, CAIC, OneD, HiCNAtra) in attenuating the 1D signal variation of chr 11-18 of MCF7. For each chromosome, the top panel shows the CNV track computed by HiCNAtra. Each grey dot represents the copy number of a bin. The red line represents the copy number track where any amplitude transition indicates a new CNV region. The second panel shows the raw (uncorrected) 1D signal. The next four panels show the post-correction 1D signal using ICE, CAIC, OneD and HiCNAtra tools. The blue line shows the moving average of the 1D signal.

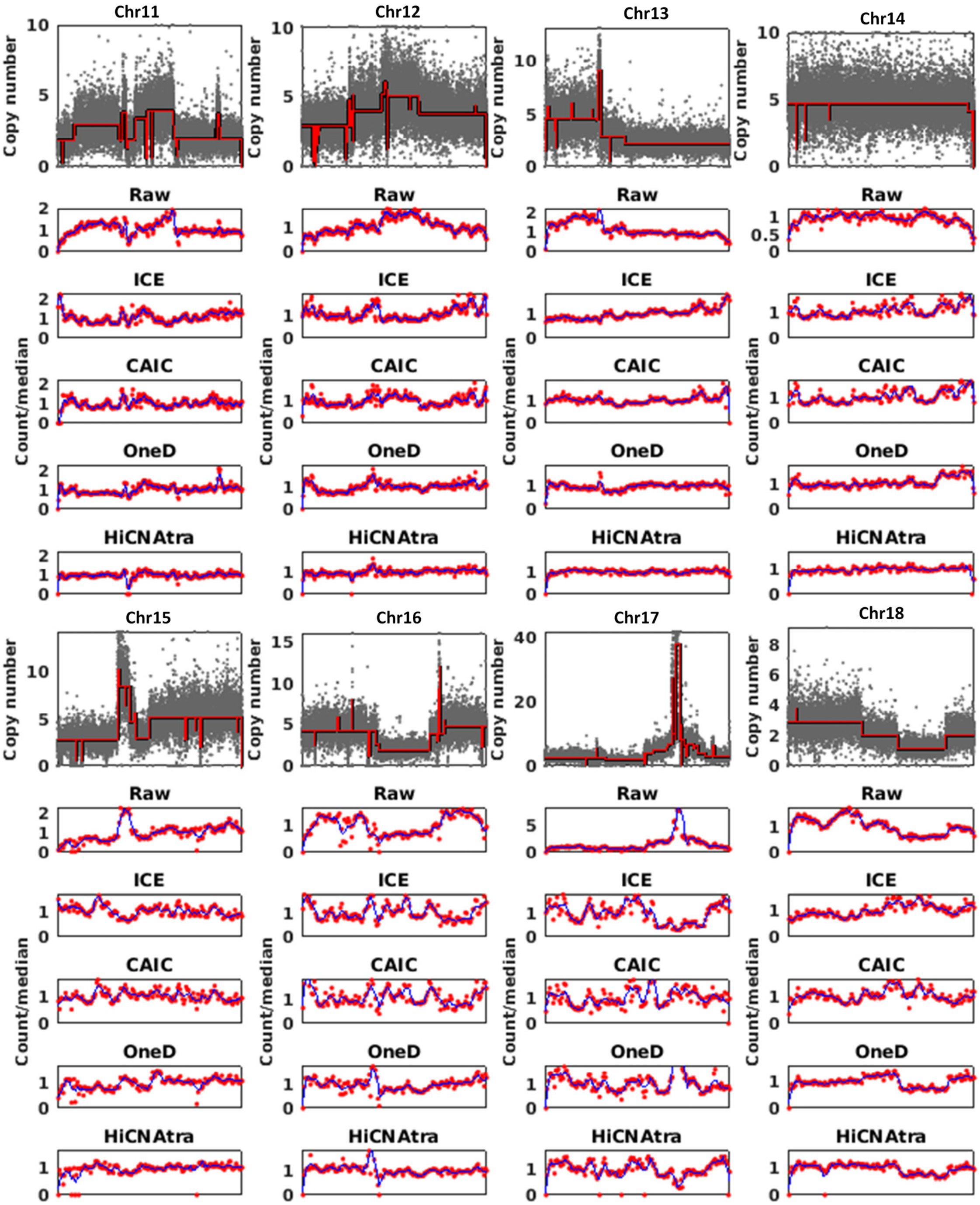
Chr11
Chr12
Chr13
Chr14
Copy number
Raw
ICE
CAIC
OneD
HiCNAtra
Count/median
Chr15
Chr16
Chr17
Chr18
Copy number
Raw
ICE
CAIC
OneD
HiCNAtra
Count/median

Figure S17

Visual comparison between HiCNAtra and OneD approaches in attenuating the 1D signal variation of PC3 chr 14 and K562 chr 3. Interaction heatmaps (500-kb bin) and 1D signals of PC3 chr 14 (left) and K562 chr 3 (right) corrected by OneD and HiCNAtra approaches. For the OneD tool, *OneD+CN* normalization module was used in all the analyses. CNV track computed by HiCNAtra is shown on top. Each grey dot represents the copy number of a bin. The red line represents the copy number track where any amplitude transition indicates a new CNV region. The second, fourth and sixth panels show the raw, OneD-corrected, and HiCNAtra-corrected contact maps respectively. Similarly, the third, fifth and seventh panels show the raw, OneD-corrected and HiCNAtra-corrected 1D signals respectively. The blue line shows the moving average of the 1D signal.

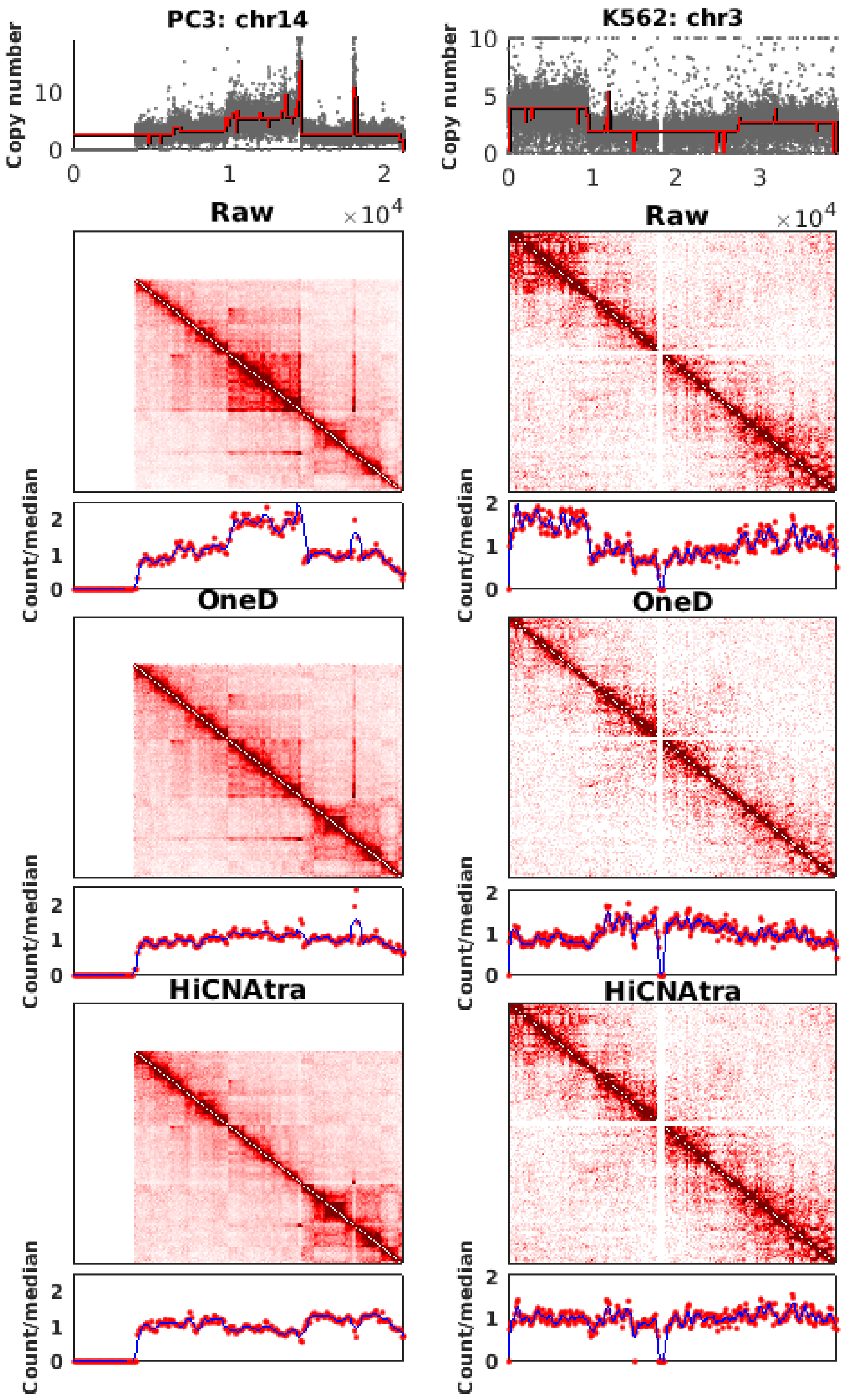

PC3: chr14
K562: chr3
Copy number
10
0
0
1
2
5
3
×10^4
Raw
OneD
HiCNAtra
Count/median

Figure S18

Visual comparison between HiCNAtra and *modified OneD* in attenuating the 1D signal variation in MCF7 chromosomes. For each chromosome (chr 1, chr 3, chr 13 and chr 15), the top panel shows the CNV track computed by HiCNAtra. Each grey dot represents the copy number of a bin. The black line represents the copy number track where any amplitude transition indicates a new CNV region. The second, fourth and sixth panels show the raw, *modified OneD*-corrected and HiCNAtra-corrected contact maps respectively. Similarly, the third, fifth and seventh panels show the raw, *modified OneD*-corrected and HiCNAtra-corrected 1D signals (red dots) respectively. The blue line shows the moving average of the 1D signal.

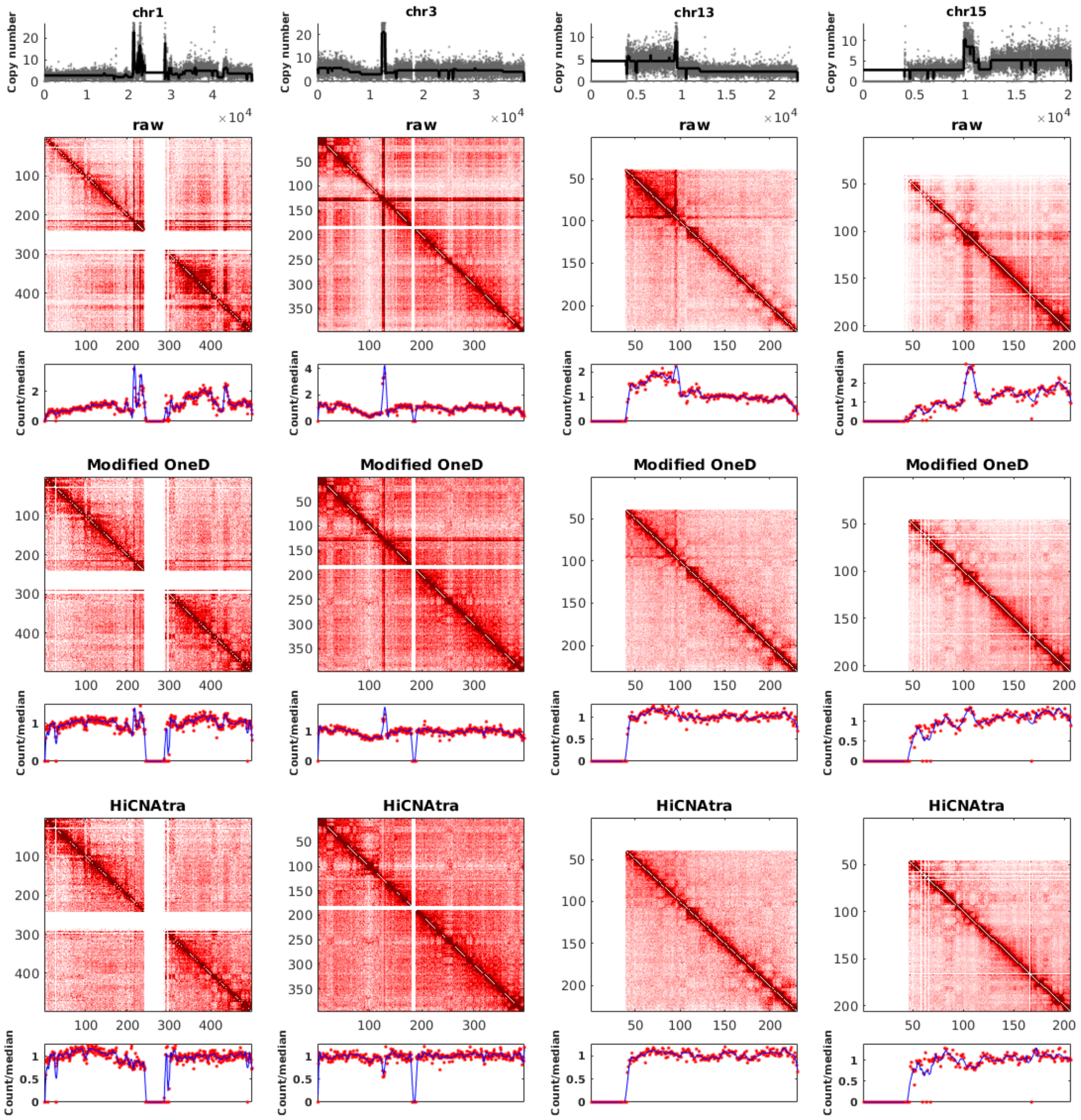

chr1
chr3
chr13
chr15
Copy number
×10^4
raw
Count/median
Modified OneD
HiCNAtra

Figure S19

Visual comparison between HiCNAtra and *modified OneD* in attenuating the 1D signal variation of LNCaP chr 10, PC3 chr 14, H69AR chr 2 and K562 chr 3. For each chromosome, the top panel shows the CNV track computed by HiCNAtra. Each grey dot represents the copy number of a bin. The black line represents the copy number track where any amplitude transition indicates a new CNV region. The second, fourth and sixth panels show the raw, *modified OneD*-corrected and HiCNAtra-corrected contact maps respectively. Similarly, the third, fifth and seventh panels show the raw, *modified OneD*-corrected and HiCNAtra-corrected 1D signals (red dots) respectively. The blue line shows the moving average of the 1D signal.

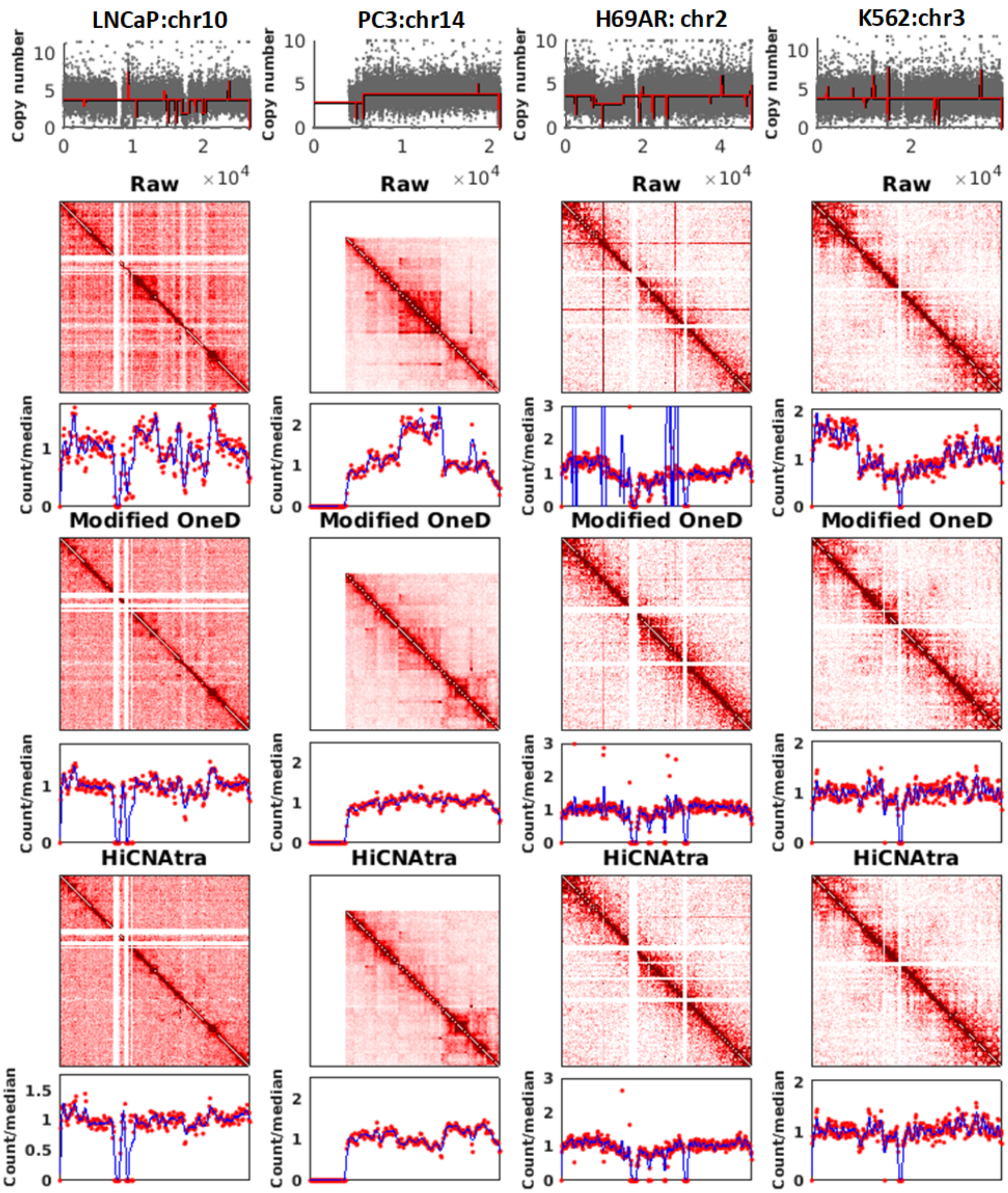

LNCaP:chr10
PC3:chr14
H69AR: chr2
K562:chr3
Copy number
Raw
×10^4
Count/median
Modified OneD
HiCNAtra

Figure S20

HiCNAtra's simultaneous approach is better than *modified OneD*'s sequential approach for correcting contact maps. (a) Bean plot of the standard deviations per chromosome of the raw (orange), *modified OneD*-corrected (green) and HiCNAtra-corrected (blue) *cis* contact maps of cancer cell lines (MCF7, LNCaP, PC3, H69AR and K562). (**b**) Bean plot showing Spearman correlations between *cis* contact frequencies and sample-dependent (CNV) and sample-independent (effective fragment length, GC-content and mappability) biases in raw, *modified OneD*-corrected (green) and HiCNAtra-corrected (blue) contact maps. Correlations are calculated across chromosomes of five cancer datasets (MCF7, LNCaP, PC3, H69AR and K562).

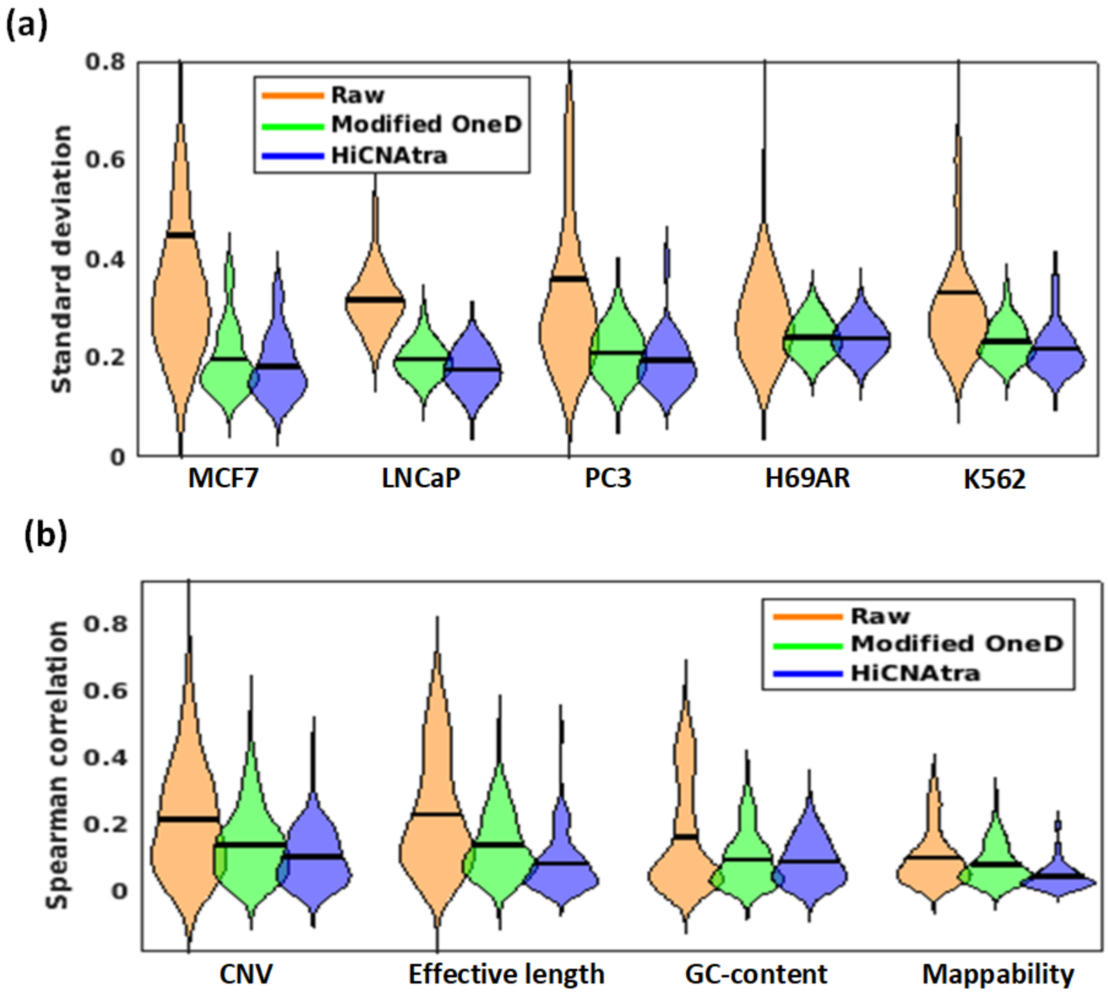
(a)
Standard deviation
0.8
0.6
0.4
0.2
0
Raw
Modified OneD
HiCNAtra
MCF7
LNCaP
PC3
H69AR
K562
(b)
Spearman correlation
0.8
0.6
0.4
0.2
0
Raw
Modified OneD
HiCNAtra
CNV
Effective length
GC-content
Mappability

Figure S21

Fragment lengths of dangling-end and extra dangling-end reads from Hi-C/3C-seq datasets. For each dataset, we approximately set the maximum molecule length (MML) as the size (in hundreds of bp) that is greater than the fragment lengths (side1-start to side2-end) of 99% of dangling-end and extra dangling-end reads. The calculated MML for GM12878 (a), IMR90 (b), MCF7 (c), LNCaP (d), PC3 (e) and H69AR (f) datasets are shown.

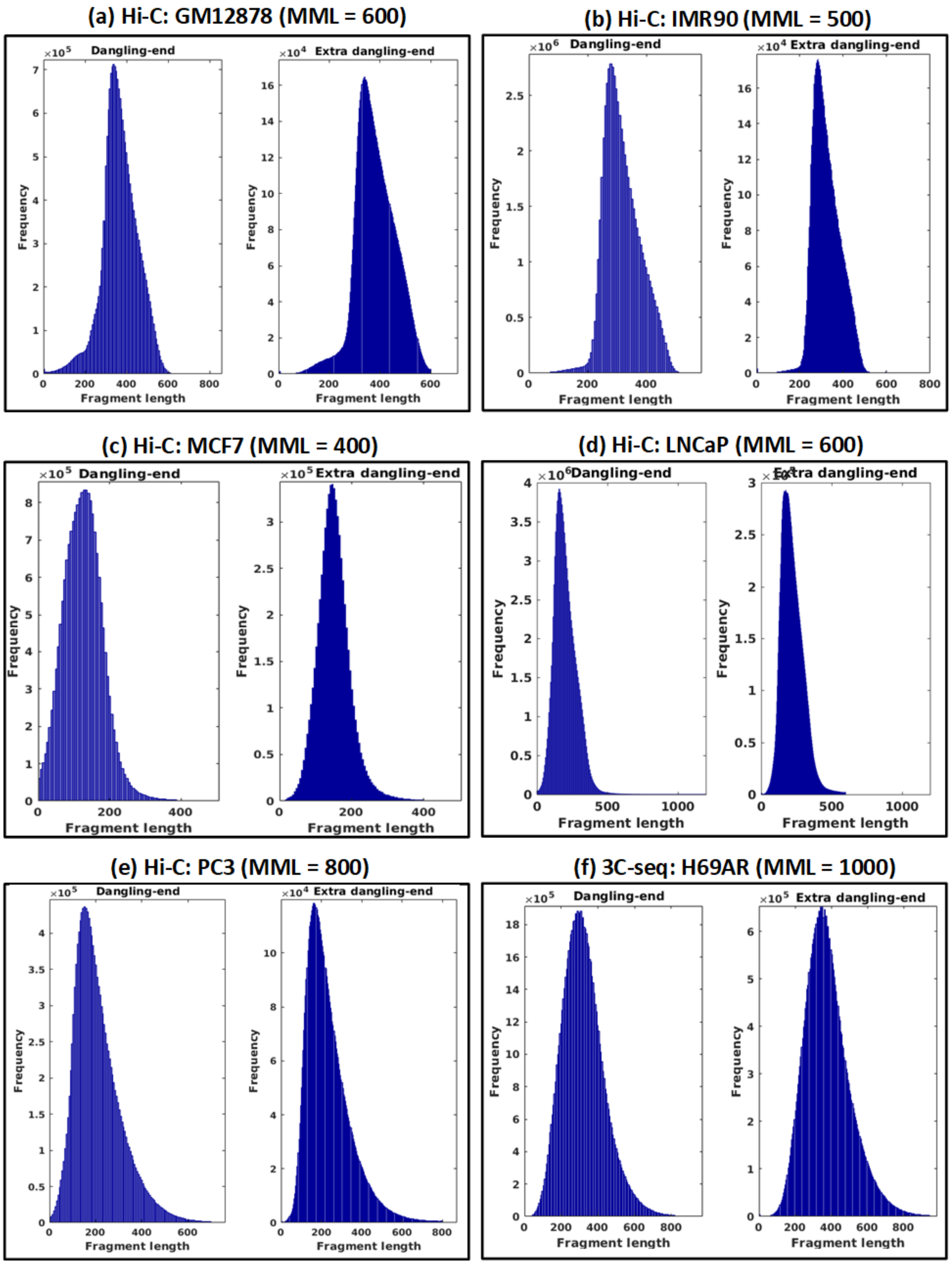
(a) Hi-C: GM12878 (MML = 600)
Dangling-end
Extra dangling-end
(b) Hi-C: IMR90 (MML = 500)
Dangling-end
Extra dangling-end
(c) Hi-C: MCF7 (MML = 400)
Dangling-end
Extra dangling-end
(d) Hi-C: LNCaP (MML = 600)
Dangling-end
Extra dangling-end
(e) Hi-C: PC3 (MML = 800)
Dangling-end
Extra dangling-end
(f) 3C-seq: H69AR (MML = 1000)
Dangling-end
Extra dangling-end
Frequency
Fragment length
